# Full-Day Physical Activity and Sedentary Behaviour Levels of Typically Developing Children and Adolescents in the Middle East: A Systematic Review

**DOI:** 10.3390/ijerph20206940

**Published:** 2023-10-19

**Authors:** Esraa Burahmah, Sivaramkumar Shanmugam, Ben Stansfield

**Affiliations:** School of Health and Life Sciences, Glasgow Caledonian University, Cowcaddens Road, Glasgow G4 0BA, UK; esraa.burahmah@gcu.ac.uk (E.B.); sivaram.shanmugam@gcu.ac.uk (S.S.)

**Keywords:** physical activity, sedentary behaviour, Middle East, children, adolescents, physical behaviour, self-report, body-worn sensor, environment, culture

## Abstract

Physical activity (PA) and sedentary behaviour (SB) are important components of physical behaviour associated with long-term health outcomes. Environmental and cultural factors may influence physical behaviour. To explore full day PA and SB in children and adolescents (2–18 years old) in the Middle East, a systematic literature review was performed including 183 journal articles. A wide range of PA and SB outcomes were reported, in some cases making synthesis of results difficult. As a consequence, results were generally reported narratively (MVPA time, total PA, SB time). Meta-regression of daily step count revealed females took 4600 fewer steps than males, with 3000 fewer steps on weekdays than weekends, and overweight individuals taking 2800 fewer steps/day. Steps decreased with age. Meta-regression for TV viewing time demonstrated an increase by 0.04 h per year of age. Even though environmental and cultural conditions may be different, PA and SB of children and adolescents in the Middle East were largely comparable to those of Europeans and North Americans. The wide range of data collection instruments used (both self-report questionnaire and body-worn devices) and heterogeneity of data made synthesis of reported data across studies very difficult, suggesting a need for greater standardisation of data collection methods.

## 1. Introduction

The World Health Organisation (WHO) recommends that, to maintain health, children and adolescents aged 5–17 years of age should engage in 60 min of moderate to vigorous physical activity (MVPA) per day, vigorous-intensity aerobic activities three times per week and limit sedentary time [1]. Both physical activity (PA) and sedentary behaviour (SB) are important components of physical behaviour as they have been independently associated with long-term health outcomes [2,3]. Establishing the volume of children and adolescents’ physical behaviour requires some form of assessment using either self-reporting (e.g., International Physical Activity Questionnaire (IPAQ) [4]), reporting by an observer (e.g., parent or carer) or the use of body-worn monitors.

The globally recognised recommendations for PA and SB provide minimum values associated with the maintenance of good health and the prevention of the development of non-communicable diseases (e.g., diabetes type II). When establishing the physical behaviour levels of groups, it is possible to assess behaviour against the minimum recommendations (e.g., % performing > 60 min of MVPA per day [5]). However, this does not provide a description of the actual volume of the behaviour. To more clearly define actual behaviour, reporting of volume indicators (e.g., actual hours per day of PA) allows evidence of behaviour time distributions to be explored.

Physical behaviour is context-specific and may vary depending on a number of environmental as well as socio-cultural factors. For example, extreme ranges of temperature will influence the amount of time that can be spent outside [6]. and cultural norms may influence types of behaviour that are acceptable [7]. Different regions of the world have different climates and different cultural norms, so it might be expected that children and adolescents exhibit different physical behaviour patterns in different regions. Additionally, groups within populations may face differing challenges to engaging with PA. For example, contextual factors may impact on males and females differently [8], or those who live in rural and urban areas [9]. 

Although there is no universally accepted definition of the region of the world considered to be the ‘Middle East’, it is possible to identify a group of countries which might be considered to have similar socio-cultural characteristics (primary Muslim, Arab) [10] and environmental characteristics. The Middle East has an environment that is characterised by very high summer temperatures, making outdoor activity challenging in some regions in the summer months [11]. Extreme temperature events in this region appear to be on the increase [12], possibly leading to even greater restrictions on PA engagement. Gender and cultural norms are highlighted as barriers to PA participation throughout the region [11]. Therefore, it is possible, due to cultural and environmental reasons, that PA and SB characteristics of children and adolescents in this region are different from those living in Europe and North America, for example. Numerous reports of physical behaviour of children and adolescents in Middle Eastern countries are available through large studies such as the CASPIAN series and through use of the ATLS instrument [13,14]. However, to date, there has been no systematic review of this material and, specifically, no extraction of daily volumes of PA and SB measures.

The aim of this systematic review of the literature was therefore to provide a summary of the daily volumes of PA and SB of children and adolescents (2–18 years of age) in the Middle East region as recorded using both self/observer-reporting and body-worn monitors. The objective was not to explore the proportion meeting physical behaviour guidelines but to characterise the absolute volume of physical behaviours.

## 2. Materials and Methods

A systematic review was conducted of published literature on daily volume of physical activity and sedentary behaviour of children and adolescents (2–18 years old) in Middle Eastern countries. The Preferred Reporting Items for Systematic Reviews and Meta-Analyses (PRISMA 2020) guidelines were used to guide implementation of the review (Supplementary Table S1).

### 2.1. Geographical Area of Interest: The Middle East

For the purposes of this review the Middle East region was defined to include countries of the Arabian Peninsula (Yemen and the Gulf Cooperation Council (GCC) countries: Bahrain, Kuwait, Oman, Qatar, Saudi Arabia, UAE) and the modern Levant area (Iraq, Israel, Jordan, Lebanon, Palestine, Syria) [15]. Additionally, areas that have historically been associated with the Middle East (Egypt, Turkey, Cyprus) were included. Cyprus is viewed as a European country; however, geographically and historically, it is located in the Middle East [16]. Although the term Middle East and North Africa (MENA) is used to define a region, an extended list of North African countries was not included in the current review due to diverging cultural and socio-economic patterns from the core Middle Eastern countries. 

### 2.2. Information Sources and Search Strategy

The Cumulative Index to Nursing and Allied Health Literature (CINAHL), Medical Literature Analysis and Retrieval System Online (MEDLINE), Web of Science and PsychINFO were used for this literature review. Both terms associated with physical activity (including ‘steps’) and sedentary behaviour (including ‘sitting’) were used alongside explicit reference to children or adolescents within the country context of interest (full search terms available in Supplementary Material S2). The search was conducted in April 2021.

### 2.3. Eligibility Criteria

Population: young people (children and adolescents) between the ages of 2 and 18 years old living in the Middle East and typically developing were the target population. Inclusion of participants above the age of 18 or below 2 years old did not exclude studies as long as the mean/mid group age for reported data was in this range. For data to be included, it was necessary for details of the age distribution to be included. As a minimum, the range of data was accepted. If data for a country (or region) of interest could not be isolated, it was not included. Only children and adolescents without any reported motor, developmental or cognitive disability were included. 

Outcome measure: to be eligible for the review, publications had to provide a measure of physical activity or sedentary behaviour which could be interpreted as a whole-day measure. 

Study design: any form of study design was accepted (e.g., cross-sectional, longitudinal, intervention, etc.) as long as original data were reported.

No restriction was placed on publication year. Primary data published in peer-reviewed English language journals were included.

### 2.4. Selection Process

Details of identified studies were imported to Mendeley reference manager (version 1.19.8), where duplicates were removed. Subsequently, references were uploaded to Rayyan [17], where titles and abstracts were reviewed against the inclusion and exclusion criteria by two reviewers independently (two from EB, BS, SS). Articles considered to be irrelevant by two reviewers were excluded at this stage; all others were taken forward.

Full texts of all papers were retrieved where possible and reviewed independently by two reviewers (two from EB, BS, SS). Where conflicts arose as to whether to include the paper, agreement was reached through discussion. Reasons for rejection were recorded and are reported via a PRISMA flow chart [18].

### 2.5. Data Extraction Process

Data were manually extracted from the papers and entered into an Excel spreadsheet. Data were checked by a second reviewer (EB, BS). Participant characteristics (sample size, M/F, age, BMI, medical conditions) were extracted. Based on possible influence on the level of physical activity and sedentary behaviour, a number of additional participant and study characteristics were extracted (country, region, urban/rural, public or private school, year of data collection, season/months of data collection). To characterise the PA or SB, additional descriptive variables were extracted (specific PA/SB definition, data collection instrument, number of weekday/weekend days of data recording).

For interventional studies, baseline data were extracted, as they represented a typical population before intervention. Where papers referred to other sources for the definition of outcomes, details were extracted where possible from the original source. All relevant PA and SB outcomes were extracted. If the children and adolescents’ condition was not mentioned, they were assumed to be typically developing.

### 2.6. Quality Assessment

The quality of all selected papers was assessed against an adapted version of the quality appraisal tool proposed by Chaabane et al. [19]. This included characteristics related to population, outcome definition, methodology, setting, timing, sampling and response rate, to give a total score out of 18 (see Supplementation Material S3). Quality assessment was performed by two reviewers and any differences resolved through discussion (EB, BS). All available data have been reported with quality score used to identify weakly reported data. However, no data were excluded based on reporting quality.

### 2.7. Synthesis Methods

The intention of the analysis was to implement meta-regression analysis of each PA or SB outcomes against a number of possible covariates, using weighting of the study data standard error. Backward stepwise selection of covariates was used to identify significant contributors to the description of the outcome of interest. Beta coefficients with 95% confidence intervals describe confidence in model fit. I^2^ was used to evaluate the heterogeneity of included study results. Where necessary, subgroup analysis was considered. SPSS version 28.0.1.1 (IBM Corporation, Armonk, NY, USA) was used for all statistical analysis. If statistical analysis was not possible, a narrative synthesis was planned.

As it has previously been demonstrated that physical behaviour may be related to various characteristics, the intention was to include the following characteristics in the meta-regression: age [20,21], gender [20,22], weight status [23,24], the nature of the educational establishment (state/public or private) [25,26], location of residence (urban or rural) [27,28] and the data collection time period (week or weekend [29], season [30,31] and year [32,33]).

#### Conversion of Reported Outcomes to Allow Standardised Presentation

In anticipation of a range of reporting methods, a uniform method of presenting a summary across data was required. To achieve this, outcomes reported in the literature were adapted as indicated in Table 1 and Table 2.

When reporting the age range, all maximum ages with fractions were used exactly as they were. However, if a range was given, the maximum ages were rounded to the next whole number, i.e., if the age range was 10–12 years, minimum age was reported as 10 years and maximum age was reported as 13. 

Bubble plots were used to illustrate relative sample size associated with each data point. Where overall sample size for a study was reported and data presented split into subcategories of study participants, for illustrative purposes if no sample size of subgroups was provided, an equal split to each sub-group was assumed.

## 3. Results

### 3.1. Study Selection

**Figure 1:**
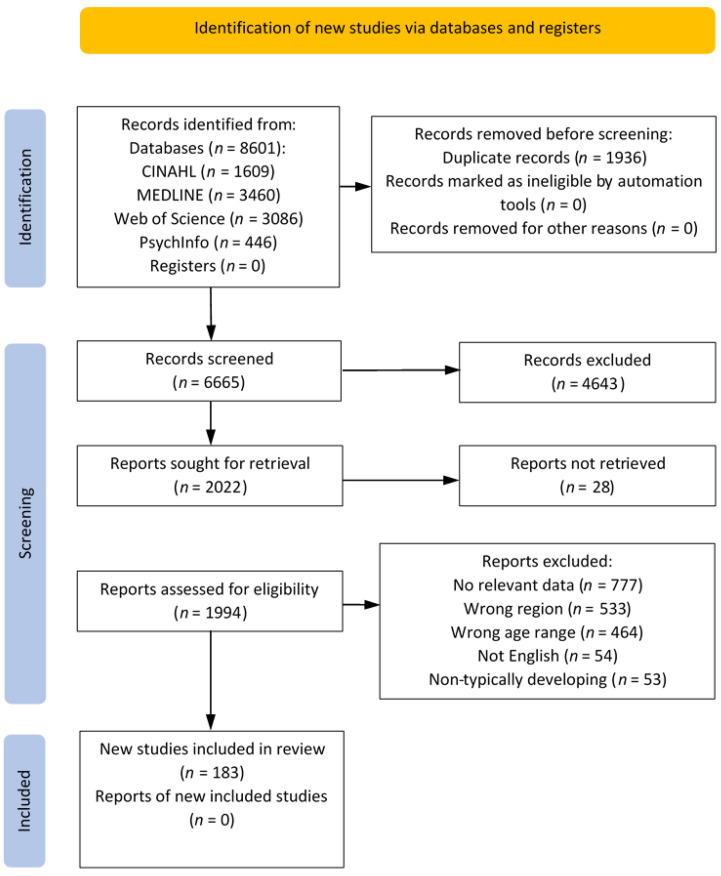
PRISMA flow chart of the number of journal articles included at each stage of the review process [18]. Within ‘Screening’, records were first screened by title and abstract, followed by retrieval of full texts and assessment of eligibility based on the full text.

After duplicates had been removed, 6665 studies were identified as relevant to the topic area (Figure 1). Following abstract and title screening, 2022 studies remained. Of these, it was possible to retrieve all but 28 full texts (there was no response from any authors for copies of full texts or additional data). Based on full text review, a substantial number of the studies (777) did not report daily measures of PA or SB, 533 did not have data specifically associated with a country of interest, 464 reported data on participants with groups of mid-age outside 2–18 years (typically young adult populations), 54 reported data not in the English language and 53 reported only data on non-typically developing children. A total of 183 individual journal article reports were included in the final data summary (a full list of all references is available in Supplementary Information Table S4) [34,35,36,37,38,39,40,41,42,43,44,45,46,47,48,49,50,51,52,53,54,55,56,57,58,59,60,61,62,63,64,65,66,67,68,69,70,71,72,73,74,75,76,77,78,79,80,81,82,83,84,85,86,87,88,89,90,91,92,93,94,95,96,97,98,99,100,101,102,103,104,105,106,107,108,109,110,111,112,113,114,115,116,117,118,119,120,121,122,123,124,125,126,127,128,129,130,131,132,133,134,135,136,137,138,139,140,141,142,143,144,145,146,147,148,149,150,151,152,153,154,155,156,157,158,159,160,161,162,163,164,165,166,167,168,169,170,171,172,173,174,175,176,177,178,179,180,181,182,183,184,185,186,187,188,189,190,191,192,193,194,195,196,197,198,199,200,201,202,203,204,205,206,207,208,209,210,211,212,213,214,215]. The eligible studies were all published after 2000 with 6, 25, 53 and 73 articles published between 2000–2004, 2005–2009, 2010–2014 and 2015–2019, respectively. Twenty six articles were published from 2020 onwards. The vast majority of study designs were cross-sectional in nature with a small number of experimental intervention studies (baseline data presented in this report) and longitudinal studies [98,169,201,205].

Of particular note in terms of reasons for exclusion was the large number of studies where reports of PA were in relation to meeting specific daily targets. Reports typically presented % of samples reaching specific PA targets. For this review, the daily volume of PA or SB was required. Percentages of participants meeting specified targets did not meet this inclusion criteria, as the specific level of PA was usually not reported. 

The number of studies and total sample size varied widely between countries (Table 3) with the largest number of studies (68) and total sample size (>100,000) being from Iran. Data by country are reported in Table 3 under various sub-categories of PA and SB. 

### 3.2. PA and SB Outcomes

The number of papers included in this review was 183. Of these, 63 reported both PA and SB, 80 reported only PA and 40 reported SB variables only. PA and SB were reported using various terminologies. The full range of reported outcomes is listed in Table 4. A total of over 1100 reportable PA or SB items were found in the 183 papers. 

All outcomes extracted from all papers are included in Supplementary Data (Table S5). The reader is referred to the Supplementary Data should they wish to extract data on the full range of variables listed in Table 4. Only those variables with sufficiently consistent definitions and for which there were suitable volumes of available data are reported here.

Of all variables reported in the literature, there was only sufficient, consistent information to warrant reporting for the following variables:

- PA: steps, MVPA (time), total PA (METs mins/day, IPAQ, and ATLS questionnaires).
- SB: TV screen time (overall and specifically for the ATLS and CASPIAN questionnaires), time (body-worn devices).

### 3.3. Study Quality

There was a wide range of study quality reported (median 11, IQR 4-13 out of a maximum score of 18) (Table 5). Generally, the quality scores suggested most studies used well-defined outcomes, although 9% were not well defined. Most studies (75%) used subjective data collection methods with only 21% using objective device-recorded outcomes. The setting was often not clearly reported with specific location only given in 34% of cases and only combined with information on urban/rural in 10% or cases. No information on time of data collection was present in 33% of cases, but year only (27%) and additionally season (39%) were reported across the majority of studies. Sampling typically included some element of random selection (68%), although response rate was often not reported (55%).

### 3.4. Sample Characteristics

Only a small number of papers (ten articles) reported data for children with mid-age less than 6 years. There were 56 articles with mid-age >6 and ≤12 years, and 117 with mid-age >12 and ≤18 years old. 

Details of participant characteristics were often absent from reports. Data were present in the following proportions across the studies: BMI (60.8%), urban/rural (41.3%), sex (78.7% identified as either M or F, remainder mixed sex), day of the week (PA/SB 88.7/69.3% identify either as weekday, weekend day or both, remainder unknown), number of days over which measurements taken (PA/SB 87.3/65.2%), year (72.2%), time of year (56.1% as either precise month or season), public/private schools (50.2%) and socio-economic status (20.1%, but often mixed). The lack of consistency of reporting of key participant characteristics made summary using meta-regression inappropriate for most variables. Additionally, data were often reported for groups of participants as averages across large age ranges (e.g., 6–13 years of age [184]).

The variables with the largest volume of data were step count (measured either using pedometers or accelerometers) and TV viewing time (measured using a range of different questionnaires). A meta-regression of these variables was performed grouping reports which did not present specific characteristics into an additional analysis category. For example, data were often presented as combined male and female data. This was considered a separate category to male and female in the meta-regression analysis. The expected effect of this was to reduce the likelihood of detecting significant effects of group characteristics on differences between groups, as including the mixed group increased the number of groups and the mixed group would fall between the other groups. A backward step-wise approach was taken to isolate significant covariates. This should be considered an exploratory analysis. 

All other data are presented in a graphical and narrative form.

### 3.5. Physical Activity Measurements

#### 3.5.1. Step Count

**Figure 2:**
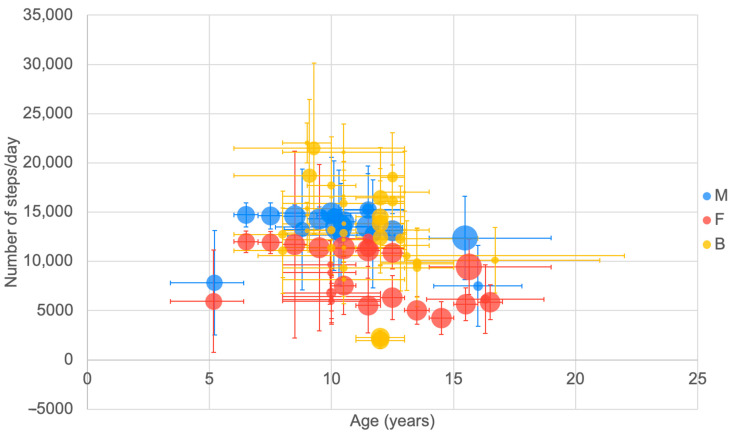
Number of reported daily steps by age. Mid age is used to present the data with range (see Table 1). Mean number of steps per day is presented ±SD (see Table 2). Bubbles indicate the relative sample size between studies. Data are presented as male (M), female (F) or both (B).

Reported step-count data were collected using a range of body-worn devices including both accelerometers and pedometers. A range of accelerometer types (SenseWear [216], Actigraph [49,66,67,123,124,145,161,168,213] ActivPAL [79]) and pedometer types (e.g., Yamax Digiwalker SW 701 [52] Omron HJ-112 [62], Kenz Lifecorder [95,194], Healer MP-22 [187], Silva [92], Oregon Scientific PE320 [78]) were used in data collection. A wide range of step-count values were reported across the age range studied (4000–23,000 steps per day) (Figure 2). 

Overall, there appeared to be a trend for females to record lower step counts than males across the full age range. However, there were numerous studies with combined results for males and females where step count was reported as being relatively high. Additionally, there appeared to be a reduction in step count with higher age. This reduction in step count with higher age was particularly evident in one large cross-sectional study where data were collected within year groups from 6–13 years [92].

##### Meta-Regression Analysis of Step Count

For the journal papers reporting step count, there was a range of missing data in terms of the covariates of interest. The year of data collection was only reported in a small number of studies and could not be included in the analysis. Socio-demographic status was rarely reported and therefore not included. Nature of schooling, either public of private, was also poorly reported and therefore not included in analysis. Sex was always reported as either M/F or both. Mid age could always be calculated and was used in the analysis. Weight status was dichotomised as normal weight (assumed status if not specified) and overweight (where specified). Day of the week was categorised as either weekend, weekday or unspecified. Urban/rural was either specified as such or in an unspecified category. Similarly, season was either summer (April–September) or winter (October–March) with an additional unspecified category.

**Figure 3:**
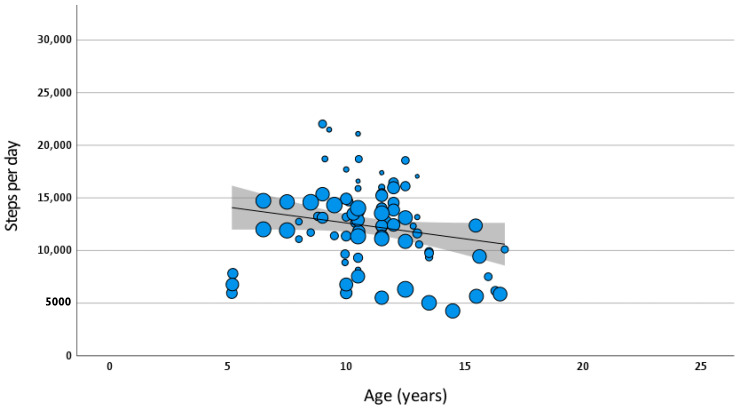
Meta-regression of steps per day by age (with adjustment for sex, weight status and day of week). Bubble size for each data point represents standard error. Prediction line and 95% confidence intervals are shown.

When all covariates (sex, weight status, day of week, age, urban/rural, season) were included in a meta-regression model of daily steps, urban/rural and season were not significant contributors. Therefore, the final model included sex, weight status, day of week and age (Table 6) (Figure 3). Age was centred at 12 years. A male of normal weight aged 12 years took on average 15,794 steps per day at the weekend. Females took 4602 fewer steps than males, being overweight was associated with 2799 fewer steps and 3098 fewer steps were taken on weekdays compared to weekends. The reports that combined both males and females appeared to show step count near to the values of males (only 212 higher on average). Where day of the week was unspecified, the step count appeared to be nearer to the weekday count than the weekend count.

Exploration of the heterogeneity of results indicated that it was not possible to assume that all studies originated from the same underlying population (I^2^ = 99.9%). Examination of visual plots of data did not suggest any obvious subgroup analysis. These results suggest that there were differences in the underlying populations from which the samples were taken that were not captured by the variables included in the analysis.

#### 3.5.2. MVPA Time (Body-Worn Device)

**Figure 4:**
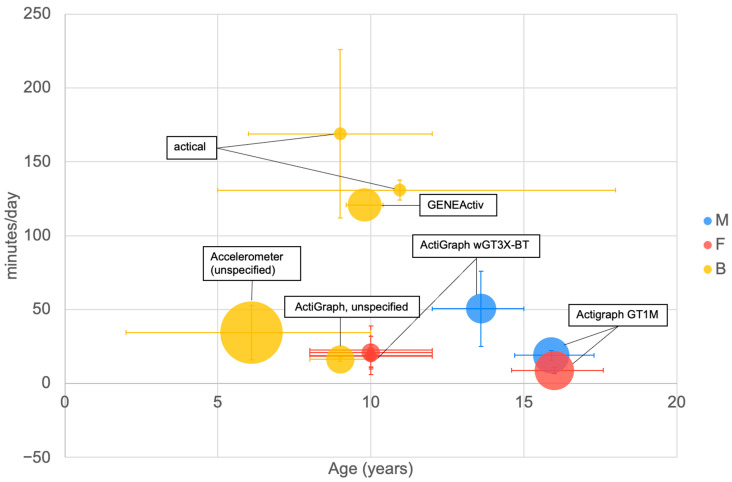
Minutes per day spent in MVPA by age measured using body-worn devices. The type of body-worn device used to measure the variable is illustrated. The largest bubble refers to a sample size of 484 [179]. See Figure 2 for explanation of plot.

A number of reports were available on the time spent in moderate to vigorous physical activity derived using body-worn devices (Figure 4). The number of minutes per day that was reported varied widely. The methods of calculating the MVPA time depended on device-specific methods and the implementation of the method used by the authors. There was no clear trend across age groups or between the sexes in terms of minutes of MVPA per day, which ranged from virtually zero up to 170 min. Variation in device wear time, definition of MVPA within signal analysis algorithms and different data sources (e.g., use of acceleration—Actigraph [57], heart rate—Actical [217]) may have influenced outcomes differently between studies.

The large sample of children with mean age of 6 years was derived as part of the IDEFICS study with an undefined body-worn accelerometer device [179]. All papers reported that body-worn devices were kept on during waking hours, except one point (130.83 min/day [113]), which reported 5 consecutive days’ wear. It appeared that values measured using similar types of body-worn devices were clustered.

#### 3.5.3. Total PA (Mets min/day) (IPAQ Questionnaire)

**Figure 5:**
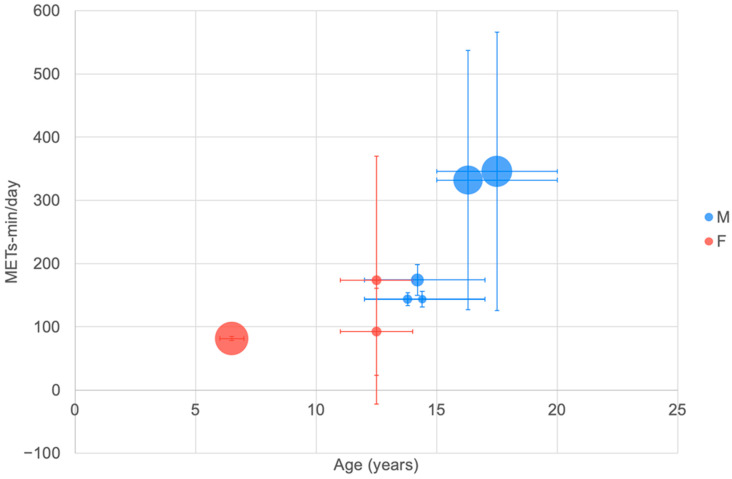
Total PA in METs min/day measured using the IPAQ by age. The largest bubble refers to a study with a sample size of 672 [34]. See Figure 2 for explanation of plot.

The International Physical Activity Questionnaire [4] was used in a number of large studies to record overall daily physical activity in METs min/day (Figure 5).

The data presented in Figure 5 represent the outcomes of large studies in Iran conducted between 2013 and 2019. Generally, males exhibited higher total PA in terms of METs min/day than females, although in the reported studies, males were generally older than females.

#### 3.5.4. Total PA (METs Min/Day) (ATLS Questionnaire)

**Figure 6:**
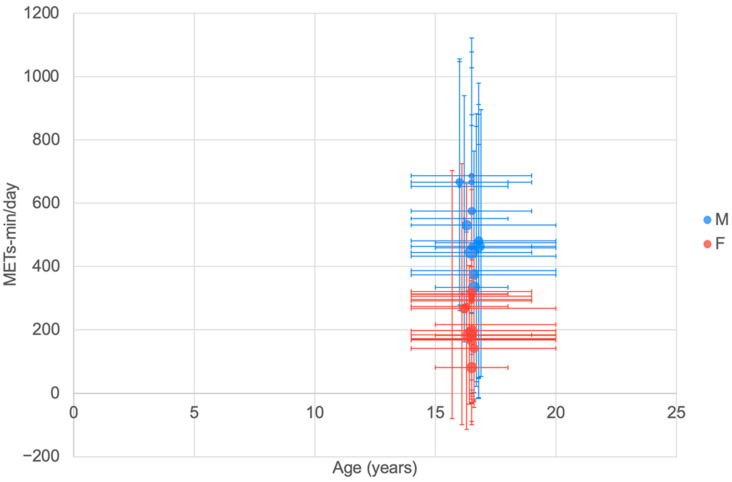
Physical activity as measured in METs min/day reported using the ATLS questionnaire by age. The largest bubble represents a sample size of 851 for girls and 797 for boys [57]. See Figure 2 for explanation of plot.

The ATLS (Arab Teens Lifestyle Study) [13] was used in a number of studies in Saudi Arabia, Kuwait, Qatar, and Jordan to report the total physical activity in METs min/day equivalents (Figure 6). Sample mean values appeared to indicate that males (400–700 METs min/day) were engaging in higher PA compared to females (100–350 METs min/day). The ATLS METs min/day values appeared to be generally higher than those reported for the IPAQ (Figure 5).

### 3.6. Sedentary Behaviour Measures

#### 3.6.1. TV Viewing Time

There were numerous reports of TV viewing time with data collected using a range of different questionnaires. A meta-regression was implemented to predict TV viewing time with covariates urban/rural, year of data collection, season (summer/winter), age, sex (male, female or both), weight normal or overweight), and day of week (weekday, weekend or unidentified).

**Figure 7:**
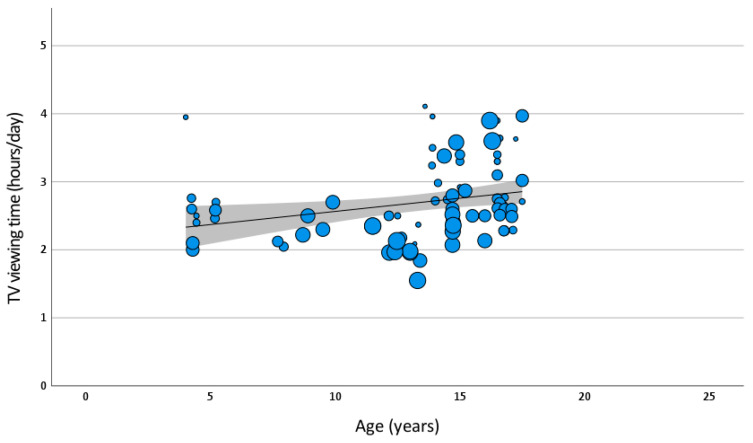
Meta-regression model of TV viewing time by age. Bubble size for each data point represents standard error. Prediction line and 95% confidence intervals are shown.

There were insufficient numbers of reports containing urban/rural (8/81) and day of week (6/81) to reasonably include these variables in the model. Again, socio-demographic status was not reported widely, or reported as mixed. Only age was a significant contributor in the prediction of volume of TV viewing time (Table 7) (Figure 7). For a 12-year-old, the average TV viewing time was 2.643 h per day. For every year older, 0.039 h more TV viewing time per day was predicted in the model.

There was extensive heterogeneity of results (I^2^ = 99.3%), suggesting that across studies the data did not originate from the same underlying population. No clear subgroup analysis was warranted. It is possible that variables not captured in this review are important explanatory factors in TV viewing time.

#### 3.6.2. Screen/TV Time (ATLS, CASPIAN)

Two data collection tools used to collect information on screen/TV time were reported in several studies. These were the ATLS [13] and CASPIAN (Childhood and Adolescence Surveillance and Prevention of Adult Non-Communicable Disease in Iran [14]) study data collection tools.

##### TV Viewing/Screen Time (ATLS)

Questions asked in the ATLS questionnaire around TV viewing and screen time were “how long per day do you watch TV and/or DVD/Video during week days?”, “how long per day do you watch TV and/or DVD/Video during weekends?”, “how long per day do you spend on the computer and/or the internet (for leisure) during week days?” and “how long per day do you spend on the computer and/or the internet (for leisure) during weekends?”.

**Figure 8:**
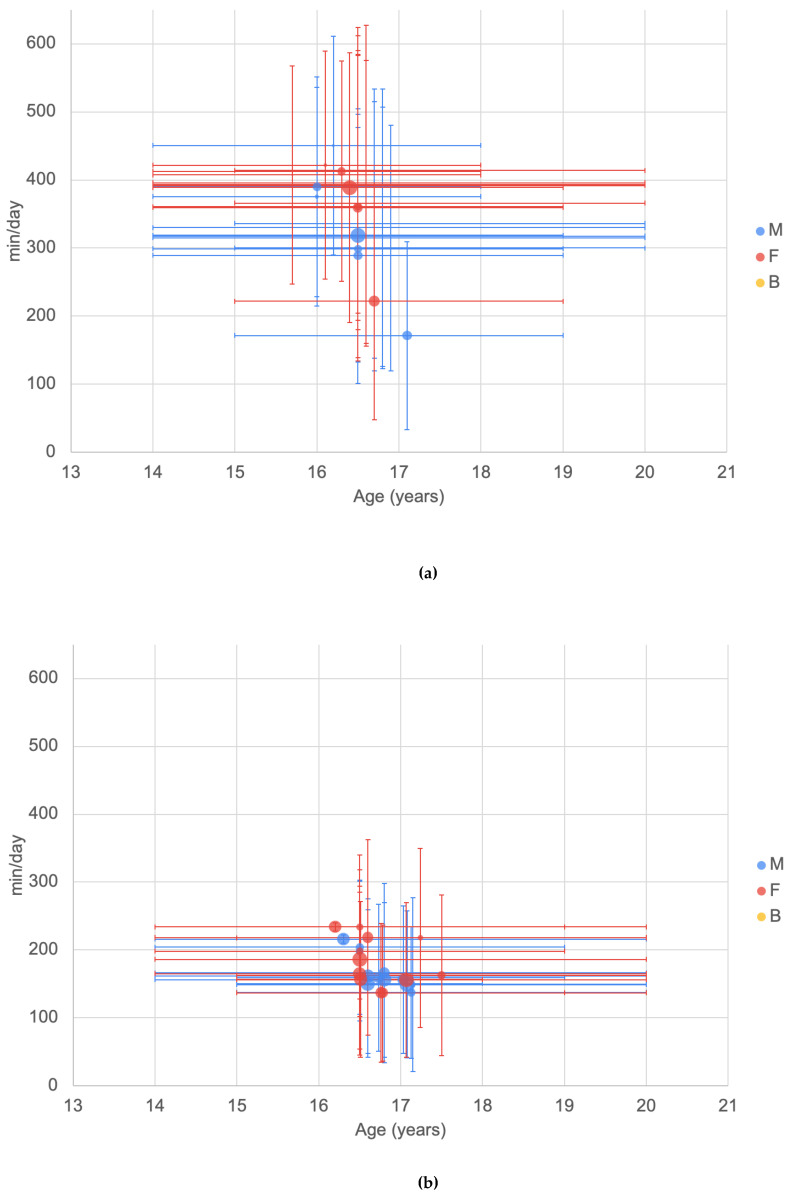
Minutes per day by age from the ATLS questionnaire of (**a**) screen time (largest bubble represents a sample size of 851 [62]); (**b**) TV viewing time (largest bubble represents a sample size of 663 [70]). See Figure 2 for explanation of plot.

For screen time and TV time reported using the ATLS questionnaire (Figure 8), two groupings of reported time (mins per day) are seen with screen time being higher than reported TV time. Reported data were predominantly derived from samples with wide age ranges. Screen time ranged from 171.6 to 450.6 min/day, and TV viewing time ranged from 136.8 to 234 min/day. There was no clear difference between males and females.

#### 3.6.3. Screen/TV Time (CASPIAN)

**Figure 9:**
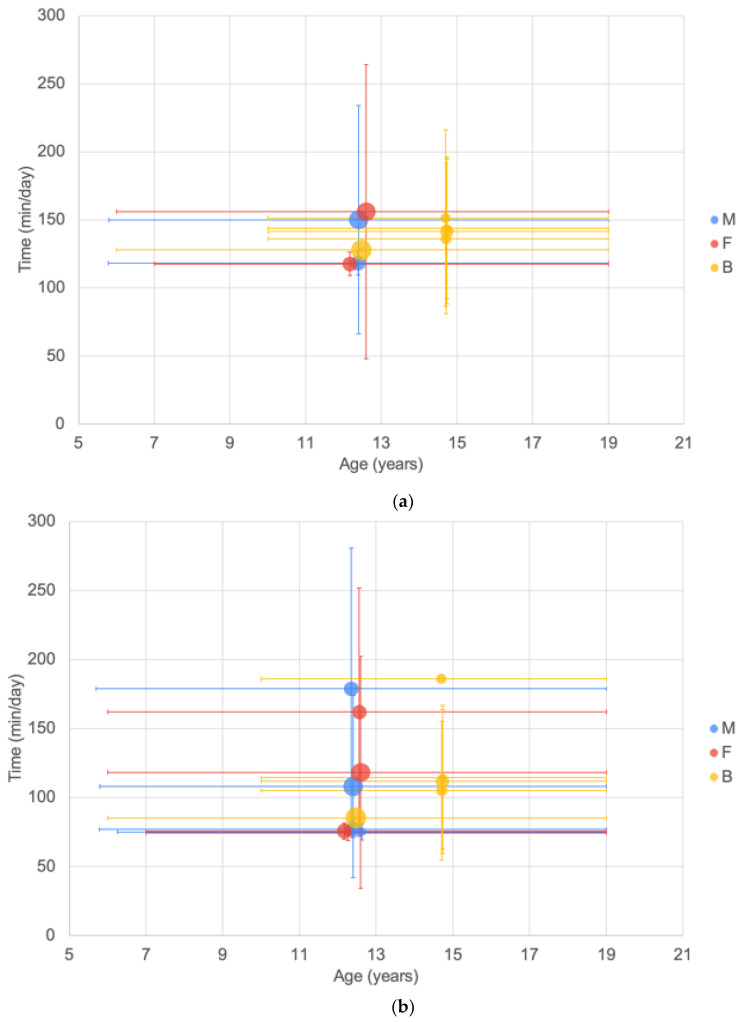
Minutes per day by age from the CASPIAN series of studies of (**a**) time spent watching a screen; (**b**) time spent watching TV. Largest bubbles represent a sample size of 13,486 [128]. See Figure 2 for explanation of plot.

For the data reported using the CASPIAN data collection tool, screen time ranged from 74.22 to 186 min/day and TV watching time from 117.6 to 151.2 min/day (Figure 9). There were insufficient data to observe trends with age or gender.

#### 3.6.4. Sedentary Time (Body-Worn Devices)

**Figure 10:**
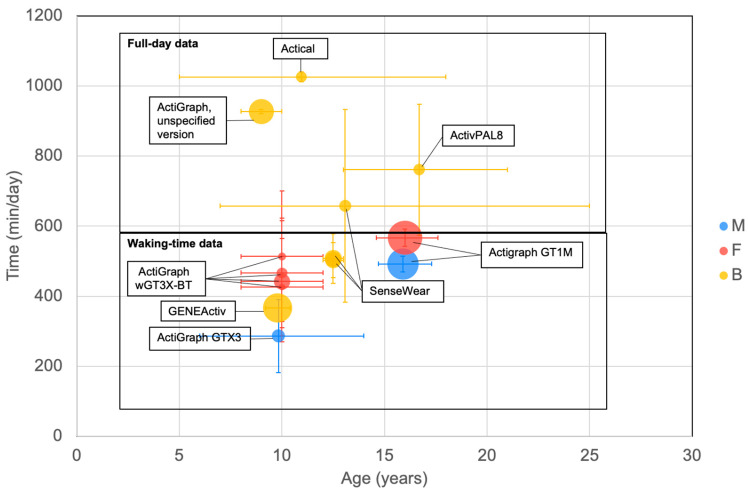
Minutes per day spent in sedentary behaviour derived using body-worn devices. The horizontal line delineates full-day data from waking-time data. Largest bubbles relate to a sample size of 189 for girls and 162 for boys [123]. See Figure 2 for explanation of plot.

There were a number of reports on the daily volume of sedentary time (mins per day) as measured using body-worn devices (Figure 10). No clear trend was observed in sedentary time. Studies detailed a range of different protocols for defining sedentary time. This included variation in device wear time (i.e., full or waking day—see Figure 10). 

All points below 600 min per day of sedentary time measured waking time only. All points above 600 measured full 24 h days. There was not a clear trend of sedentary behaviour measured using a body-worn device with age.

## 4. Discussion

This systematic literature review was performed to gather previously reported information on the daily volumes of PA and SB in children and adolescents 2–18 years old in Middle Eastern countries. This was done to provide a characterisation of physical behaviour in this area of the world that might be considered to have broadly similar environmental and cultural factors. Any measurements of PA and SB were considered acceptable for inclusion as long as they were representative of whole-day physical behaviour, derived using either objective body-worn devices or subjective self- or observer-reported questionnaires. A total of 183 journal articles reporting PA or SB were identified, reporting a range of outcome measures. Of all outcome measures reported, it was only possible to present a statistical analysis of daily step count and TV viewing time with other outcomes being described graphically and narratively due to small numbers of reports.

The search terms of the literature review were kept intentionally broad to ensure all reports of physical activity and sedentary behaviours were included. 

The area of interest, the Middle East, was defined based on a range of countries where cultural influences and environmental influences might lead to similar constraints on and opportunities for PA and SB. While the authors acknowledge that the extent of the boundaries of the area considered to constitute the Middle East are not well defined, the choice of countries to include was justified based on historical, cultural and environmental considerations. To allow the reader to interpret results based on isolated countries, Supplementary Data is provided with a full list of all variables from all available literature (Supplementary Data S5).

The review identified a substantial body of literature reporting daily PA or SB measures (183 journal articles). However, reports were concentrated mainly in Iran (68 publications), Turkey (37) and Saudi Arabia (27) with none reporting data from Bahrain, Syria or Yemen. There were a number of large cohort studies associated with these concentrations of reporting (e.g., ATLS: Saudi Arabia (Al-Khobar, Jeddah, Riyadh, Al-Ahsa), Kuwait, Qatar, Iraq, Jordan, Oman; CASPIAN: Iran). The larger cohort studies provided evidence of random sampling across regions, and therefore, reported data should be representative of national trends in physical behaviour for the age groups studied. However, in other countries, the sample sizes were smaller and must be considered to be less representative of the broader national populations of children (e.g., Egypt, Iraq, Palestine, Qatar, UAE). An additional constraint on the reported data is that reports often grouped children and adolescents across broad age ranges [49,62,65], making identification of trends with age difficult.

The variables reported were of numerous types with many different quantities defined (Table 4). The intention to present meta-regression analysis could only be achieved for step count and TV viewing time due to the small number of studies on each PA or SB measure. It was possible to present a number of PA and SB measures graphically. Even within this limited selection of variables, there were several data collection methods used which made comparison between study outcomes difficult. For example, MVPA was measured using various different technologies (Figure 4) (e.g., incorporating data from accelerometry, body temperature [218], heart rate [219]). For identification of SB, the surrogate of TV viewing or screen viewing was reported in several studies. However, different definitions were used in different studies (e.g., ATLS, CASPIAN), making comparison between studies challenging.

A simple quality assessment was performed to identify key components of study reporting. Either unclear reporting or use of non-standardised outcome measures (without clear validity) was present in 47% of studies, highlighting difficulties in comparing outcomes between studies. This points to a need for the adoption of standardised and validated body-worn device outcomes or self-report measures across studies to allow data gathering and exploration of changes in PA and SB across ages and over time. Ambiguity in the exact questions asked in questionnaires concerning sedentary time (TV viewing, screen viewing, etc.) were particularly problematic for comparison of sedentary outcomes. Perhaps this difficulty has been compounded by a lack of foresight by researchers in anticipating changes in technology. Not all studies reported whether the setting was urban/rural or reported the time of year. Without reporting of such demographic and season data, it is difficult to understand differences between study results. While a majority of studies (68%) reported an element of randomisation in selection of their sample population, there was limited reporting of response rate, potentially introducing bias into the reported data.

An attempt was made to extract information on participant BMI, sex, socio-demographic status, day of week data was recorded for, and if study participants attended public or private schools. It is possible that all of these variables may influence physical behaviour, but they were not reported in all studies, again making inter-study comparison difficult.

### 4.1. Reports of Physical Activity

PA was reported using a wide range of quantities. This included time spent in postures (e.g., standing) and the number of steps taken per day. These are relatively unambiguous concepts that can be measured using body-worn devices. Additionally, PA was reported in relation to energy expenditure, either as daily energy expenditure or as time spent at set energy expenditure levels (e.g., moderate or vigorous intensity). The determination of these quantities was device- or questionnaire-dependent, causing difficulties with comparison between studies.

#### 4.1.1. Stepping Activity

The most enduring measure of physical activity performance appears to be step count. Studies reported step count evaluation using body-worn devices including pedometer and other accelerometer-based devices (Figure 2). It was possible to perform a meta- regression analysis of step count, suggesting age, sex, weight status and day of the week were important in describing variance (Table 6). However, there was a high level of heterogeneity between study results, suggesting that there were differences in the underlying populations from which samples were taken. The step counts reported were broadly similar to those reported for other regions of the world: Male weekend daily step count for 12-year-olds (15,845) was higher compared to similarly aged children in America [220]. However, day of the week is often not specified, and there was an indication of ~3000 fewer steps being taken on weekdays. Females took fewer steps (−4602 per day), similar to observations in other parts of the world, for example in Australia [221] (10,463 for boys and 8940 for girls.), in Canada [222] (12,100 for boys and 10,300 for girls) and in Finland [8] (10,824 for boys and 9040 for girls). Additionally, those who were characterised as over-weight took fewer steps (−2799 per day), again similar to other regions, e.g., UK [223] and Ireland [224]. 

Based on the identified relationships between step count and gender, body weight, day of the week and age, there is evidence (although weak) to support similar patterns of activity in the Middle East to those in Europe and North America (as referenced previously). This would suggest that the effect of these factors on step count are similar even though the cultural and environmental contexts are different. It is possible therefore that the underlying reasons for differences between sub-groups of children and adolescents may be the same across the world, including the impact of differences in social interactions for boys and girls, the association of excessive body weight with activity levels, the importance of day of the week and the general decline in activity (stepping) with age. These similarities may suggest that components of interventions to increase stepping activity (e.g., enhanced social support [225]; enhanced psychological support [226]) could be similar across contexts, including the Middle East.

#### 4.1.2. MVPA Time (Body-Worn Devices)

The reported values of MVPA time (Figure 4) highlight differences between different devices (e.g., actical, ActiGraph). It appeared that different wear time protocols and different algorithms for identifying MVPA time made values incomparable. Standardisation of measurements is required before data can be reasonably synthesised.

#### 4.1.3. Total PA (METs min/day) IPAQ and ATLS

The IPAQ and ATLS have been used in large cross-sectional studies to determine daily PA time as METS min/day (Figure 5 and Figure 6). Unfortunately, the data reported did not cover the age range of interest well and the IPAQ results appeared to be generally lower than the ATLS outcomes. Overall, however, it is possible to observe a higher total PA level for males than females. The total PA measured using IPAQ showed that males engage in more total PA compared to females, in agreement with Bauman et al. [227], which was conducted in 20 countries.

### 4.2. Reports of Sedentary Behaviour

#### 4.2.1. Screen/TV Time

There were several different methods used to assess screen time. A common method was to ask questions about TV screen time (‘TV watching’, ‘TV viewing’, ‘TV time’). Other combinations of screen time, including video, computer games and mobile phone, were reported (see Supplementary Material S5). However, it was difficult to compare between studies as technological changes reduced the relevance of chronological comparison. This evolution of terminology added a layer of complexity to comparing not only changing questions, but also changing meaning of the same question. For example, ‘screen time’, has developed from including only TV viewing to including progressively video, gaming devices, computers and mobile phone screens. To compound difficulties with exploration of these changing definitions, numerous studies did not report the year of data collection.

When TV viewing time was taken in isolation, a meta-regression analysis suggested that only age was an important predictor, indicating an increase of 0.039 (95% CI: 0.008, 0.069) hours per day per year of age. However, due to high levels of heterogeneity in study outcomes and no clear sub-group analysis pathway, these results must be treated with a high level of caution.

While TV viewing time is highlighted here as a possible daily SB quantity, a study conducted on teenagers in the UK suggests that TV viewing time does not reflect full-day sedentary time [228,229]. Also, McGrane Minton et al. [230] suggest not using full-day sitting time and TV viewing time interchangeably when measuring SB. TV viewing can therefore only be considered a partial surrogate for SB. 

Unfortunately, due to the various definitions used in questionnaires, it was not possible to robustly examine total screen time across all studies. The changing self-report questions concerning screen-based device usage tend to reflect changing habits. For example, although Mullan [231] report a 30 min per day increased use of screen-based devices among 8–18-year-old children in the UK from 2000 to 2015, this was attributed to an increase in boys playing video games. 

In a scoping review by Thomas et al. (2019), it was highlighted that total screen time (TV viewing, computer use, and playing video games) among 5–18-year-olds increased over time, but within this group, TV viewing declined a little. Thomas et al. [232] also showed an increase in overall screen time over a 4-year period (2010–2014) for Australian children aged 10–15 (TV viewing, computer use, and social networking and online communication). Differences in cultural norms and environmental factors may have made changes in the Middle East different to those seen in other continents [19,233,234]. Unfortunately, the inconsistent use of terminology between studies made exploration of changes with chronological time impossible.

#### 4.2.2. SB Body-Worn Devices

The disparate results of sedentary behaviour time per day reported using body-worn devices point to a need for a standardisation of measurement in this area. 

### 4.3. Limitations

There is no clearly identified region which constitutes the Middle East. However, for the current review, countries were included where it was anticipated cultural and environmental factors may be similar. 

This review did not include an analysis of the grey literature. It is possible that, for example, governmental reports may add further insight into PA and SB levels in this region.

It was not possible to gain access to original data sets. No authors responded to requests for additional information. Gaining further details on study cohorts, timing of data collection and details of specific data collection instruments not included in reports would have been helpful in informing outcome analysis.

The use of a wide range of data collection instruments that were often poorly defined and lacked consistency of application between studies made synthesis of results difficult across most outcome measures.

It was common for studies to report outcomes for grouped results across wide age ranges; this may have masked age-related changes.

The meta-regression analyses reported identified heterogeneity of data and should be considered to be exploratory in nature.

## 5. Conclusions

There is extensive reporting of PA and SB in children and adolescents across the Middle East through small-scale studies, but also through relatively large-scale cross-sectional studies with randomised selection of participants. However, there is a lack of standardisation of outcome measures, making synthesis of findings across studies difficult. The absence of longitudinal studies prevents definitive conclusions on changes of behaviour with age, as shifting patterns of behaviour (e.g., screen viewing) may be the underlying cause of changes rather than age.

Where sufficient data were available, it was observed that those children and adolescents who were male, of normal weight and younger appeared to undertake more PA in terms of stepping activity. More stepping activity was undertaken at weekends. This was reinforced for questionnaire-based outcomes of PA energy expenditure in terms of METs min/day, where males tended to report more daily PA.

TV viewing time as a surrogate for sedentary behaviour suggested an increase with age. There was insufficient data of objectively measured sedentary time to allow synthesis and analysis of the influence of covariates. 

Generally, trends in PA and SB between males and females were similar to those found in European/North American studies, although there may be greater differences in the Middle Eastern region. The observation of similar differences between sexes in different cultures and environments points to the possibility that there may be common behavioural, social and psychological factors affecting relative physical behaviour levels. This may imply that similar intervention strategies can be used across regions to enhance physical behaviour for improved long-term health.

Overall, while there were a large number of reports of PA and SB, the outcomes reported were disparate and confounded by changing behaviour in regard to engagement with technology (SB measured using TV/screen viewing). There is a need for further standardisation of measurement to ensure synthesis of report findings is possible in the future.

## Figures and Tables

**Table 1:** Conversions of reported age variables to allow standardised presentation.

Available Data	Mid Age	Age Min	Age Max	+Error Bar	−Error Bar
Range and mean	Mean	range min	Range max (if whole number round to next whole number)	(Age max) − (Mid age)	(Mid age) − (Age min)
Range only	(Age min) + (Age max − Age min)/2				
Mean and SD	Mean	(Mid age) − (2 × SD)	(Mid age) + (2 × SD)	2 × SD	2 × SD

SD = standard deviation, min = minimum, max = maximum.

**Table 2:** Conversions of outcomes to mean and SD values where necessary to allow standardised presentation. Non-parametric distribution characteristics were converted to the equivalent normal distribution characteristics to allow presentation of data within the same graphical display.

Original PA or SB Outcome	Converted Outcome
Median (using equivalent normal distribution)	Mean
IQR/1.35 (using equivalent normal distribution)	SD
95%CI/4 (assumed 95% CI ≈ 4 × SD)	SD
Range/6 (assumed 99% CI ≈ 6 × SD)	SD
SE × square root of sample size (Based on definition of SE)	SD

IQR = interquartile range, SD = standard deviation, CI = confidence interval, SE = standard error.

**Table 3:** The number of studies, sample size, and PA and SB measurements reported by country.

Country *	Number of Studies	Total Sample Size (Duplicate Samples Combined)	PA Measure Category					SB Measure Category					
			Steps 1	Kcal/ Total EE 3	Counts 4	Time 5	METs 8	TV/DVD/Video 1	Computer/ Computer Games/Internet 2	Combination of Screen Time 3	Total Screen Time/ Electronic Use 4	Time 6	METs 8
Cyprus	13	4482	6	0	0	5	0	4	1	2	4	1	0
Egypt	2	363	0	2	1	0	0	0	0	0	0	0	0
Iran	68	101,611	1	5	1	28	19	15	14	7	11	8	1
Iraq	3	913	0	0	1	1	1	1	0	1	0	0	0
Israel	8	5223	0	0	0	3	0	1	1	0	3	2	0
Jordan	4	4358	0	0	0	0	2	3	1	0	1	0	0
Kuwait	4	1292	0	1	0	3	1	1	1	0	0	2	0
Lebanon	5	7923	0	0	0	2	0	1	1	1	1	1	0
Oman	6	2114	0	0	0	4	0	0	0	3	2	1	0
Palestine	1	378	0	0	0	1	0	0	0	0	0	0	0
Qatar	1	1161	0	0	0	0	1	0	0	0	1	0	0
Saudi Arabia	27	10,627	5	3	0	8	10	9	7	4	6	7	0
Turkey	37	18,208	7	7	0	13	8	4	1	1	2	11	0
UAE	3	666	0	1	0	1	0	1	0	0	0	3	0

* There were no sources including Bahrain, Syria or Yemen. One source was mixed Palestine, Jordan and Israel. EE = energy expenditure, METs = metabolic equivalents.

**Table 4:** Variables reported in the literature characterising daily volumes of PA or SB. Both objective (device-measured) and subjective (self/observer-reported) data types are listed.

	Objective or Subjective	Variable	Sub-Categories	Base Units *
PA	Objective	Steps		Steps
		Energy expenditure	TEE, DEE, AEE	Kcal or kJ
		Counts		Counts
		Time		Mins
	Subjective	METs	Light, Moderate, Vigorous, Moderate + Vigorous	MET mins
		Time	Light, Moderate, Vigorous, Moderate + Vigorous Exercise/sport	Mins
		Postures	Upright, transitions	Mins or number
SB	Objective	Time		Mins
	Subjective	Screen-based activity time	Either one or a combination of the following: TV/DVD/video/video games/computer/computer games/internet/smart phone/tablet	Mins
		Sedentary-based activity time	Sedentary time/physical inactivity/doing home-work/sitting/reading books/desk work	Mins
		METs		MET mins

* Base units used in description of variable (per day or per week). TEE = Total energy expenditure, DEE = Daily energy expenditure, AEE = Activity energy expenditure, and MET = Metabolic equivalent, mins = minutes.

**Table 5:** Quality assessment of included studies (percentage per score).

	Quality Assessment Criteria					
Grade	Outcome Definition	Data Collection Instrument	Setting	Timing	Sampling	Response Rate
0	not defined	not defined	not defined	not stated	not stated	not defined
1	unclear non-standard	non-validated	region/city (+1)		self-selected or unclear	<50%
2	clear non-standard	validated	specific location (+1)	Year	clear non-random	50–79%
3	standard	objective	urban/rural (+1)	year and season	random (by location or within location)	80%+
Grade	Percentage per criterion					
0	1	4	10	33	15	55
1	8	24	46	1	7	1
2	39	51	34	27	10	11
3	53	21	10	39	68	33

**Table 6:** Meta-regression model of step count including sex, weight status, day of week and age as covariates. Note: age centred at 12 years.

Parameter		B Coefficient	95%CI	*p* Value
Intercept *		15,794	13,928, 17,663	*p* < 0.001
Sex	Female	−4602	−6131, −3074	*p* < 0.001
	Male + Female	212	−1263, 1686	0.776
Weight status	Overweight	−2799	−4832, −767	0.001
Day of week = week day	Week day	−3098	−4850, −1347	0.001
	Unspecified	−2282	−3985, −578	0.009
Age		−260	−513, −8	0.044

* Intercept value for male, normal weight, weekend, age of 12 years. CI = confidence interval.

**Table 7:** Meta-regression model of TV viewing time (hours/day) including age as a covariate. Note: age centred at 12 years.

Parameter	B Coefficient	95%CI	*p* Value
Intercept *	2.643	2.515, 2.771	<0.001
Age	0.039	0.008, 0.069	0.014

* Intercept value for age 12 years old. CI = confidence interval.

## Data Availability

To enquire about access to the data used to develop outcomes in this paper, please contact the lead author or refer to Supplementary Material Table S5.

## Supplementary Materials

The following supporting information can be downloaded at https://www.mdpi.com/article/10.3390/ijerph20206940/s1, Table S1: PRISMA Checklist; Table S2: Search terms used within the CINAHL, MEDLINE, Web of Science and PsychoINFO databases; Table S3: Quality assessment criteria with definitions used to facilitate scoring; Table S4: Reference list of all papers included in the review; Table S5: Extracted PA and SB data from all studies identified.
