# Supplementary material for: Full-Day Physical Activity and Sedentary Behaviour Levels of Typically Developing Children and Adolescents in the Middle East: A Systematic Review
Source: Int J Environ Res Public Health. 2023 Oct 19;20(20):6940. doi: 10.3390/ijerph20206940 (PMC10606092; doi:10.3390/ijerph20206940)
Supplement: Supplementary file 1 [file ijerph-20-06940-s001.zip › Burahmah PA SB review S1-S4 v2.pdf]

Supplementary Material

Table S1. PRISMA 2020 checklist

Section and Topic	Item #	Checklist item	Location where item is reported
**TITLE**			
Title	1	Identify the report as a systematic review.	Title
**ABSTRACT**			
Abstract	2	See the PRISMA 2020 for Abstracts checklist.	Abstract
**INTRODUCTION**			
Rationale	3	Describe the rationale for the review in the context of existing knowledge.	End Introduction
Objectives	4	Provide an explicit statement of the objective(s) or question(s) the review addresses.	End Introduction
**METHODS**			
Eligibility criteria	5	Specify the inclusion and exclusion criteria for the review and how studies were grouped for the syntheses.	Section 2.3
Information sources	6	Specify all databases, registers, websites, organisations, reference lists and other sources searched or consulted to identify studies. Specify the date when each source was last searched or consulted.	Section 2.2
Search strategy	7	Present the full search strategies for all databases, registers and websites, including any filters and limits used.	Table S2
Selection process	8	Specify the methods used to decide whether a study met the inclusion criteria of the review, including how many reviewers screened each record and each report retrieved, whether they worked independently, and if applicable, details of automation tools used in the process.	Section 2.4
Data collection process	9	Specify the methods used to collect data from reports, including how many reviewers collected data from each report, whether they worked independently, any processes for obtaining or confirming data from study investigators, and if applicable, details of automation tools used in the process.	Section 2.5
Data items	10a	List and define all outcomes for which data were sought. Specify whether all results that were compatible with each outcome domain in each study were sought (e.g. for all measures, time points, analyses), and if not, the methods used to decide which results to collect.	Section 2.5
	10b	List and define all other variables for which data were sought (e.g. participant and intervention characteristics, funding sources). Describe any assumptions made about any missing or unclear information.	Section 2.7
Study risk of bias assessment	11	Specify the methods used to assess risk of bias in the included studies, including details of the tool(s) used, how many reviewers assessed each study and whether they worked independently, and if applicable, details of automation tools used in the process.	Section 2.6
Effect measures	12	Specify for each outcome the effect measure(s) (e.g. risk ratio, mean difference) used in the synthesis or presentation of results.	Section 2.7
Synthesis methods	13a	Describe the processes used to decide which studies were eligible for each synthesis (e.g. tabulating the study intervention characteristics and comparing against the planned groups for each synthesis (item #5)).	Section 2.7

Section and Topic	Item #	Checklist item	Location where item is reported
	13b	Describe any methods required to prepare the data for presentation or synthesis, such as handling of missing summary statistics, or data conversions.	Section 2.7
	13c	Describe any methods used to tabulate or visually display results of individual studies and syntheses.	Section 2.7
	13d	Describe any methods used to synthesize results and provide a rationale for the choice(s). If meta-analysis was performed, describe the model(s), method(s) to identify the presence and extent of statistical heterogeneity, and software package(s) used.	Section 2.7
	13e	Describe any methods used to explore possible causes of heterogeneity among study results (e.g. subgroup analysis, meta-regression).	Section 2.7
	13f	Describe any sensitivity analyses conducted to assess robustness of the synthesized results.	NA
Reporting bias assessment	14	Describe any methods used to assess risk of bias due to missing results in a synthesis (arising from reporting biases).	NA
Certainty assessment	15	Describe any methods used to assess certainty (or confidence) in the body of evidence for an outcome.	Section 2.7
**RESULTS**			
Study selection	16a	Describe the results of the search and selection process, from the number of records identified in the search to the number of studies included in the review, ideally using a flow diagram.	Figure 1
	16b	Cite studies that might appear to meet the inclusion criteria, but which were excluded, and explain why they were excluded.	Section 3.1
Study characteristics	17	Cite each included study and present its characteristics.	Table S4/S5
Risk of bias in studies	18	Present assessments of risk of bias for each included study.	Table 5
Results of individual studies	19	For all outcomes, present, for each study: (a) summary statistics for each group (where appropriate) and (b) an effect estimate and its precision (e.g. confidence/credible interval), ideally using structured tables or plots.	Table S4
Results of syntheses	20a	For each synthesis, briefly summarise the characteristics and risk of bias among contributing studies.	NA
	20b	Present results of all statistical syntheses conducted. If meta-analysis was done, present for each the summary estimate and its precision (e.g. confidence/credible interval) and measures of statistical heterogeneity. If comparing groups, describe the direction of the effect.	Table 6/7
	20c	Present results of all investigations of possible causes of heterogeneity among study results.	NA
	20d	Present results of all sensitivity analyses conducted to assess the robustness of the synthesized results.	Narrative sections 3.5/3.6
Reporting biases	21	Present assessments of risk of bias due to missing results (arising from reporting biases) for each synthesis assessed.	NA
Certainty of evidence	22	Present assessments of certainty (or confidence) in the body of evidence for each outcome assessed.	As appropriate sections

Section and Topic	Item #	Checklist item	Location where item is reported
			3.5/3.6
**DISCUSSION**			
Discussion	23a	Provide a general interpretation of the results in the context of other evidence.	Sections 4.1/4.2
	23b	Discuss any limitations of the evidence included in the review.	Sections 4.1/4.2/4.3
	23c	Discuss any limitations of the review processes used.	Section 4.3
	23d	Discuss implications of the results for practice, policy, and future research.	Conclusion section
**OTHER INFORMATION**			
Registration and protocol	24a	Provide registration information for the review, including register name and registration number, or state that the review was not registered.	NA
	24b	Indicate where the review protocol can be accessed, or state that a protocol was not prepared.	Not prepared
	24c	Describe and explain any amendments to information provided at registration or in the protocol.	NA
Support	25	Describe sources of financial or non-financial support for the review, and the role of the funders or sponsors in the review.	Funding section
Competing interests	26	Declare any competing interests of review authors.	Conflict of Interest section
Availability of data, code and other materials	27	Report which of the following are publicly available and where they can be found: template data collection forms; data extracted from included studies; data used for all analyses; analytic code; any other materials used in the review.	Data Availability Statement

Table S2. Search terms used within the CINAHL, MEDLINE, Web of Science and PsychoINFO databases.

Search terms
Characteristics of interest:
“physical activit*” OR sedentary OR (MH “Sedentary Behavior”) OR sitting OR “physical inactivit*” OR “step count”
Population:
AND child* OR (MH “Child”) OR (MH “Child, Preschool”) OR *adolescent* OR (MH “Adolescent”) OR *teenager*
Country/location:
AND Bahrain OR (MH “Bahrain”) OR Cyprus OR (MH “Cyprus”) OR Egypt OR (MH “Egypt”) OR Iran OR (MH “Iran”) OR Iraq OR (MH “Iraq”) OR Israel OR Jordan OR (MH “Jordan”) OR Kuwait OR (MH “Kuwait”) OR Lebanon OR “MH “Lebanon”) OR Oman OR (MH “Oman”) OR Palestine OR Qatar OR (MH “Qatar”) OR Saudi Arabia OR (MH “Saudi Arabia”) OR Syria OR (MH “Syria”) OR Turkey OR (MH “Turkey”) OR United Arab Emirates OR (MH “United Arab Emirates”) OR Yemen OR (MH “Yemen”) OR “Gulf countr*” OR “Middle East” OR (MH “Middle East”) OR GCC OR Arab

Table S3. Quality assessment criteria with definitions used to facilitate scoring (adapted from Chaabane et al (2020)).

Criteria's name	Criteria's scoring<br>(Max score of 3/ criteria)	Criteria's definition	Remarks
0. Population characteristics<br><br>Nationality, type of population, age groups	For inclusion in the review the journal article had to include information that would allow all criteria to be met.	The following characteristics were specified:<br>a) Nationality<br>b) Population type (typically developing)<br>c) Age group	If nationality was not stated the country of residence was used. If the country was not stated, the country in which the research committee board gave approval was assumed as the country.<br>If no population type was stated, the children were assumed to be typically developing.<br>Age group could either mean the age range, or the mean of age.
1. Outcome definition<br><br>Physical activity/Sedentary behaviour definition in the study	0 Not defined	No definition	
	1 Unclear non-standard definition	Any incomplete /partial definition of the outcome.	Screen time is unclear definition
	2 Clear non-standard definition	**PA:** The stated definition of PA is clear (time and duration) but not a daily volume of all PA. Example might be 'exercise in last week'.<br>**SB:** Any clear definition (time and duration) of only one type of SB using mean time/day or week (e.g. mean time spent watching TV only), but not total daily sedentary behaviour. Must be/involve sitting.	
	3 Standard definition. Clearly stated definition of	**PA:** Any measure of daily PA including: Steps, time in MVPA/MPA/VPA, total minutes spend in PA, TEE, PAL on a	

	PA/SB. Units expressed per day in recognized units: min or hrs/d, METs/day.	typical day<br>**SB:** Minutes spent in SB on a typical day. – all sedentary behaviour, not just category.<br>Any waking, non-standing, behaviour with an energy expenditure of ≤1.5 METs.	
2. Measurement methodology<br><br>Physical activity instrument or used items	0 Not defined	The used instrument was not defined	
	1 A non-validated questionnaire (subjective)	Use of a non-validated and non-standard questionnaire	
	2 Validated questionnaire (subjective)	Use of a validated standard PA or SB instrument	We trusted the studies when stating the validity and reliability of the outcome measures used.
	3 A gold standard (objective)	Used an objective instrument, such as, accelerometer or pedometer	
3. Setting<br><br>Setting	0 Not defined/ not clearly defined	No definition	
	3 Clearly defined<br>(1 point for each characteristic)	The following characteristics were specified:<br>a) Region/city (1 point)<br>b) Specific location, e.g. hospital, school (1 point)<br>c) Urban or rural (1 point)	For the location, for confidentiality purposes the name of the school/hospital shouldn't be stated. Urban and rural, only given a point when stated deliberately, no assumptions made
4. Timing	0 Not defined	Data collection time not stated (years of data collection, season of data collection)	Only giving a season/month without a year was scored zero.
	2 Years defined	Data collection year(s) stated.	

Years/season of data collection	3 Clearly defined years and seasons	Data collection year(s) and season(s) (months) stated	
5. Sampling<br><br>Sampling method	0 Not defined	Not stated	
	1 Unclear	Self-selection or unclear method	If stated convenient sample or not stated at all
	2 Non-random sampling	Clearly defined non-random sampling method (e.g. mechanism of selecting school/class/participants)	Clear description of how sample recruited in relation to wider population.
	3 Random sampling	Clearly defined random sampling (random at level of country region or city or school)	If stated an element of randomisation, which could represent the population, or stated a stratified multistage sampling
6. Response rate<br><br>Response rate (%)	0 Not defined	The response rate was not reported for the study population generating the specific extracted data.<br>e.g. only the overall response rate and the males and female’s response rate are not stated.	
	1 <49%	Low response rate	
	2 50-79%	Acceptable response rate in this context	
	3 >=80%	Standard response rate	

Table S4. Reference list of all papers included in the review.

Rayyan ID number	Reference
664537285	Abasi, M. H., Eslami, A. A., Rakhshani, F., & Shiri, M. (2016a). A self-efficacy questionnaire regarding leisure time physical activity: Psychometric properties among Iranian male adolescents. *Iranian Journal of Nursing and Midwifery Research*, *21*(1), 20–28. https://doi.org/10.4103/1735-9066.174751
664537249	Abasi, M. H., Eslami, A. A., Rakhshani, F., & Shiri, M. (2016b). Development and psychometric properties of a self-regulation scale about leisure time physical activity in Iranian male adolescents. *Iranian Journal of Nursing and Midwifery Research*, *21*(2), 183–190. https://doi.org/10.4103/1735-9066.178246
664536008	Abdulaziz, J. S., & Hassan, M. K. (2019). Nutritional status of children and adolescents with haemophilia in Basra, Iraq. *Haemophilia: The Official Journal of the World Federation of Hemophilia*, *25*(6), e353–e360. https://doi.org/10.1111/hae.13837
664536361	Abiri, B., Sarbakhsh, P., & Vafa, M. (2019). Prevalence of overweight, obesity, and associated risk factors in healthy female adolescents in Tehran, Iran. *Central Asian Journal of Global Health*, *8*(1). https://doi.org/10.5195/cajgh.2019.413
664536766	Ahmed, H. S., Khalid, M. E. M., Osman, O. M., Ballal, M. A., & Al-Hashem, F. H. (2016). The association between physical activity and overweight and obesity in a population of children at high and low altitudes in Southwestern Saudi Arabia. *Journal of Family & Community Medicine*, *23*(2), 82–87. https://doi.org/10.4103/2230-8229.181011
664534737	Akbulut, G., Yildirim, M., Sanlier, N., van Stralen, M. M., Acar-Tek, N., Bilici, S., Brug, J., de Meij, J. S. B., Gezmen-Karadag, M., Koksal, E., Oenema, A., Singh, A. S., te Velde, S. J., Yildiran, H., & Chinapaw, M. J. M. (2014). Comparison of energy balance-related behaviours and measures of body composition between Turkish adolescents in Turkey and Turkish immigrant adolescents in the Netherlands. *Public Health Nutrition*, *17*(12), 2692–2699. https://doi.org/10.1017/S1368980013003388
664535353	Akman, M., Akan, H., Izbirak, G., Tanriöver, Ö., Tilev, S. M., Yildiz, A., Tektaş, S., Vitrinel, A., & Hayran, O. (2010). Eating patterns of Turkish adolescents: A cross-sectional survey. *Nutrition Journal*, *9*, 67. https://doi.org/10.1186/1475-2891-9-67
664540104	Aktürk, S., Büyükavcı, R., & Aktürk, Ü. (2019). Relationship between musculoskeletal disorders and physical inactivity in adolescents. *Journal of Public Health*, *27*(1), 49–56. https://doi.org/10.1007/s10389-018-0923-7
664537911	Al Barwani, S., Al Abri, M., Al Hashmi, K., Al Shukeiry, M., Tahlilkar, K., Al Zuheibi, T., Al Rawas, O., & Hassan, M. O. (2001). Assessment of aerobic fitness and its correlates in Omani adolescents using the 20-metre shuttle run test: A pilot study. *Journal for Scientific Research. Medical Sciences*, *3*(2), 77–80.
664540288	Al Yazeedi, B. (2018). *Childhood Obesity and Family Influence on Children's Nutrition Intake, Physical Activity Patterns, and BMI Z-scores in Oman*. ProQuest Dissertations Publishing.
664535963	Alaca, N., & Yüksel, M. (2021). Comparison of physical functions and psychosocial conditions between adolescents with pectus excavatum, pectus carinatum and healthy controls. *Pediatric Surgery International*, *37*(6), 765–775. https://doi.org/10.1007/s00383-021-04857-7
664534253	Alamolhoda, M., Heydari, S. T., Ayatollahi, S. M. T., Tabrizi, R., Akbari, M., & Ardalan, A. (2020). A multivariate multilevel analysis of the risk factors associated with anthropometric indices in Iranian mid-adolescents. *BMC Pediatrics*, *20*(1), 191. https://doi.org/10.1186/s12887-020-02104-x
664538742	Albarwani, S., Al-Hashmi, K., Al-Abri, M., Jaju, D., & Hassan, M. O. (2009). Effects of overweight and leisure-time activities on aerobic fitness in urban and rural adolescents. *Metabolic Syndrome and Related Disorders*, *7*(4), 369–374. https://doi.org/10.1089/met.2008.0052
664536958	Alghadir, A. H., Gabr, S. A., & Al-Eisa, E. (2016). Effects of Physical Activity on Trace Elements and Depression Related Biomarkers in Children and Adolescents. *Biological Trace Element Research*, *172*(2), 299–306. https://doi.org/10.1007/s12011-015-0601-3
664537014	Alghadir, A. H., Gabr, S. A., & Iqbal, Z. A. (2015). Effects of sitting time associated with media consumption on physical activity patterns and daily energy expenditure of Saudi school students. *Journal of Physical Therapy Science*, *27*(9), 2807–2812. https://doi.org/10.1589/jpts.27.2807
664536995	Alghadir, A. H., Gabr, S. A., & Rizk, A. A. (2018). Physical Fitness, Adiposity, and Diets as Surrogate Measures of Bone Health in Schoolchildren: A Biochemical and Cross-Sectional Survey Analysis. *Journal of Clinical Densitometry: The Official Journal of the International Society for Clinical Densitometry*, *21*(3), 406–419. https://doi.org/10.1016/j.jocd.2017.12.006

664535513	Alghadir, A. H., Iqbal, Z. A., & Gabr, S. A. (2020). Differences among Saudi and Expatriate Students: Body Composition Indices, Sitting Time Associated with Media Use and Physical Activity Pattern. *International Journal of Environmental Research and Public Health*, *17*(3), 832. https://doi.org/10.3390/ijerph17030832
664534802	Al-Ghamdi, M. A., Lanham-New, S. A., & Kahn, J. A. (2012). Differences in vitamin D status and calcium metabolism in Saudi Arabian boys and girls aged 6 to 18 years: Effects of age, gender, extent of veiling and physical activity with concomitant implications for bone health. *Public Health Nutrition*, *15*(10), 1845–1853. https://doi.org/10.1017/S1368980011003612
664537409	Al-Hazzaa, H. M. (2007). Pedometer-determined physical activity among obese and non-obese 8- to 12-year-old Saudi schoolboys. *Journal of Physiological Anthropology*, *26*(4), 459–465. https://doi.org/10.2114/jpa2.26.459
664534660	Al-Hazzaa, H. M., Abahussain, N. A., Al-Sobayel, H. I., Qahwaji, D. M., & Musaiger, A. O. (2011). Physical activity, sedentary behaviors and dietary habits among Saudi adolescents relative to age, gender and region. *The International Journal of Behavioral Nutrition and Physical Activity*, *8*, 140. https://doi.org/10.1186/1479-5868-8-140
664535280	Al-Hazzaa, H. M., Abahussain, N. A., Al-Sobayel, H. I., Qahwaji, D. M., & Musaiger, A. O. (2012). Lifestyle factors associated with overweight and obesity among Saudi adolescents. *BMC Public Health*, *12*, 354. https://doi.org/10.1186/1471-2458-12-354
664536834	Al-Hazzaa, H. M., Alahmadi, M. A., Al-Sobayel, H. I., Abahussain, N. A., Qahwaji, D. M., & Musaiger, A. O. (2014). Patterns and determinants of physical activity among Saudi adolescents. *Journal of Physical Activity & Health*, *11*(6), 1202–1211. https://doi.org/10.1123/jpah.2012-0427
664536594	Al-Hazzaa, H. M., Alhussain, M. H., Alhowikan, A. M., & Obeid, O. A. (2019). Insufficient Sleep Duration And Its Association With Breakfast Intake, Overweight/Obesity, Socio-Demographics And Selected Lifestyle Behaviors Among Saudi School Children. *Nature and Science of Sleep*, *11*, 253–263. https://doi.org/10.2147/NSS.S225883
664536887	Al-Hazzaa, H. M., Al-Nakeeb, Y., Duncan, M. J., Al-Sobayel, H. I., Abahussain, N. A., Musaiger, A. O., Lyons, M., Collins, P., & Nevill, A. (2013). A cross-cultural comparison of health behaviors between Saudi and British adolescents living in urban areas: Gender by country analyses. *International Journal of Environmental Research and Public Health*, *10*(12), 6701–6720. https://doi.org/10.3390/ijerph10126701
664537247	Al-Hazzaa, H. M., & Al-Rasheedi, A. A. (2007). Adiposity and physical activity levels among preschool children in Jeddah, Saudi Arabia. *Saudi Medical Journal*, *28*(5), 766–773.
664538147	Al-Hazzaa, H. M., Al-Sobayel, H. I., Abahussain, N. A., Qahwaji, D. M., Alahmadi, M. A., & Musaiger, A. O. (2014). Association of dietary habits with levels of physical activity and screen time among adolescents living in Saudi Arabia. *Journal of Human Nutrition and Dietetics: The Official Journal of the British Dietetic Association*, *27 Suppl 2*, 204–213. https://doi.org/10.1111/jhn.12147
664537553	Al-Hazzaa, H. M., Al-Sobayel, H. I., & Musaiger, A. O. (2011). Convergent validity of the Arab Teens Lifestyle Study (ATLS) physical activity questionnaire. *International Journal of Environmental Research and Public Health*, *8*(9), 3810–3820. https://doi.org/10.3390/ijerph8093810
664534427	Al-Hazzaa, H. M., Musaiger, A. O., Abahussain, N. A., Al-Sobayel, H. I., & Qahwaji, D. M. (2014). Lifestyle correlates of self-reported sleep duration among Saudi adolescents: A multicentre school-based cross-sectional study. *Child: Care, Health and Development*, *40*(4), 533–542. https://doi.org/10.1111/cch.12051
664536186	Alhusaini, A. A., Ali Al-Walah, M., Melam, G. R., & Buragadda, S. (2017). Pedometer-determined physical activity levels of healthy children and children with Down's syndrome. *Somatosensory & Motor Research*, *34*(4), 219–225. https://doi.org/10.1080/08990220.2017.1415880
664535406	Alhusaini, A. A., Buragadda, S., & Melam, G. (2020). Associations among Body Mass Index, sedentary behavior, physical activity, and academic performance in schoolchildren. *The Journal of Sports Medicine and Physical Fitness*, *60*(12), 1551–1557. https://doi.org/10.23736/S0022-4707.20.10482-1
664535412	Alhusaini, A. A., Melam, G. R., & Buragadda, S. (2020). Cross-Cultural Variation in BMI, Sedentary Behavior, and Physical Activity in International School Girls Residing in Saudi Arabia. *International Journal of Environmental Research and Public Health*, *17*(6), 2057. https://doi.org/10.3390/ijerph17062057
664535430	Aliss, E. M., Sutaih, R. H., Kamfar, H. Z., Alagha, A. E., & Marzouki, Z. M. (2020). Physical activity pattern and its relationship with overweight and obesity in saudi children. *International Journal of Pediatrics & Adolescent Medicine*, *7*(4), 181–185. https://doi.org/10.1016/j.ijpam.2020.03.007

664535680	Aljuhani, O., & Sandercock, G. (2019). Contribution of Physical Education to the Daily Physical Activity of Schoolchildren in Saudi Arabia. *International Journal of Environmental Research and Public Health*, *16*(13), 2397. https://doi.org/10.3390/ijerph16132397
664534402	Al-Kutbe, R., Payne, A., de Looy, A., & Rees, G. A. (2017). A comparison of nutritional intake and daily physical activity of girls aged 8-11 years old in Makkah, Saudi Arabia according to weight status. *BMC Public Health*, *17*(1), 592. https://doi.org/10.1186/s12889-017-4506-2
664534486	Allafi, A., Al-Haifi, A. R., Al-Fayez, M. A., Al-Athari, B. I., Al-Ajmi, F. A., Al-Hazzaa, H. M., Musaiger, A. O., & Ahmed, F. (2014). Physical activity, sedentary behaviours and dietary habits among Kuwaiti adolescents: Gender differences. *Public Health Nutrition*, *17*(9), 2045–2052. https://doi.org/10.1017/S1368980013002218
664536870	Al-Nakeeb, Y., Lyons, M., Collins, P., Al-Nuaim, A., Al-Hazzaa, H., Duncan, M. J., & Nevill, A. (2012). Obesity, physical activity and sedentary behavior amongst British and Saudi youth: A cross-cultural study. *International Journal of Environmental Research and Public Health*, *9*(4), 1490–1506. https://doi.org/10.3390/ijerph9041490
664536892	Al-Nuaim, A. A., Al-Nakeeb, Y., Lyons, M., Al-Hazzaa, H. M., Nevill, A., Collins, P., & Duncan, M. J. (2012). The Prevalence of Physical Activity and Sedentary Behaviours Relative to Obesity among Adolescents from Al-Ahsa, Saudi Arabia: Rural versus Urban Variations. *Journal of Nutrition and Metabolism*, *2012*, 417589. https://doi.org/10.1155/2012/417589
664536797	Al-Sobayel, H., Al-Hazzaa, H. M., Abahussain, N. A., Qahwaji, D. M., & Musaiger, A. O. (2015). Gender differences in leisure-time versus non-leisure-time physical activity among Saudi adolescents. *Annals of Agricultural and Environmental Medicine: AAEM*, *22*(2), 344–348. https://doi.org/10.5604/12321966.1152091
664540156	Altintaş, A., & Aşçi, F. H. (2008). Physical self-esteem of adolescents with regard to physical activity and pubertal status. *Pediatric Exercise Science*, *20*(2), 142–156. https://doi.org/10.1123/pes.20.2.142
664540175	Altıntaş, A., Aşçı, F. H., Kin-İşler, A., Güven-Karahan, B., Kelecek, S., Özkan, A., Yılmaz, A., & Kara, F. M. (2014). The role of physical activity, body mass index and maturity status in body-related perceptions and self-esteem of adolescents. *Annals of Human Biology*, *41*(5), 395–402. https://doi.org/10.3109/03014460.2013.857721
664534680	Amini, M., Djazayery, A., Majdzadeh, R., Taghdisi, M.-H., Sadrzadeh-Yeganeh, H., Abdollahi, Z., Hosseinpour-Niazi, N., Chamari, M., & Nourmohammadi, M. (2016). A School-Based Intervention to Reduce Excess Weight in Overweight and Obese Primary School Students. *Biological Research for Nursing*, *18*(5), 531–540. https://doi.org/10.1177/1099800416654261
664534882	Amiri, P., Jalali-Farahani, S., Zarkesh, M., Barzin, M., Kaviani, R., & Ahmadizad, S. (2014). Reliability and validity of the Iranian version of the QAPACE in adolescents. *Quality of Life Research: An International Journal of Quality of Life Aspects of Treatment, Care and Rehabilitation*, *23*(6), 1797–1802. https://doi.org/10.1007/s11136-014-0625-8
664534557	Ardestani, M., Niknami, S., Hidarnia, A., & Hajizadeh, E. (2015). Predictors of Physical Activity among Adolescent Girl Students Based on the Social Cognitive Theory. *Journal of Research in Health Sciences*, *15*(4), 223–227.
664534625	Ardestani, M. S., Niknami, S., Hidarnia, A., & Hajizadeh, E. (2016). Psychometric properties of the Social Cognitive Theory questionnaire for physical activity in a sample of Iranian adolescent girl students. *Eastern Mediterranean Health Journal = La Revue De Sante De La Mediterranee Orientale = Al-Majallah Al-Sihhiyah Li-Sharq Al-Mutawassit*, *22*(5), 318–326. https://doi.org/10.26719/2016.22.5.318
664534611	Ardic, A., & Erdogan, S. (2017). The effectiveness of the COPE healthy lifestyles TEEN program: A school-based intervention in middle school adolescents with 12-month follow-up. *Journal of Advanced Nursing*, *73*(6), 1377–1389. https://doi.org/10.1111/jan.13217
664534201	Aviram, R., Harries, N., Shkedy Rabani, A., Amro, A., Nammourah, I., Al-Jarrah, M., Raanan, Y., Hutzler, Y., & Bar-Haim, S. (2019). Comparison of Habitual Physical Activity and Sedentary Behavior in Adolescents and Young Adults With and Without Cerebral Palsy. *Pediatric Exercise Science*, *31*(1), 60–66. https://doi.org/10.1123/pes.2017-0285
664534237	Azabdaftari, F., Jafarpour, P., Asghari-Jafarabadi, M., Shokrvash, B., & Reyhani, P. (2020). Unrestricted prevalence of sedentary behaviors from early childhood. *BMC Public Health*, *20*(1), 255. https://doi.org/10.1186/s12889-020-8346-0
664538415	Azadbakht, L., Akbari, F., Qorbani, M., Esmaeil Motlagh, M., Ardalan, G., Heshmat, R., Daneshzad, E., & Kelishadi, R. (2019). Dinner consumption and cardiovascular disease risk factors among a nationally representative sample of Iranian adolescents: The CASPIAN-III Study. *Journal of Cardiovascular and Thoracic Research*, *11*(2), 138–146. https://doi.org/10.15171/jcvtr.2019.24

664534457	Bagherniya, M., Mostafavi Darani, F., Sharma, M., Maracy, M. R., Allipour Birgani, R., Ranjbar, G., Taghipour, A., Safraian, M., & Keshavarz, S. A. (2018). Assessment of the Efficacy of Physical Activity Level and Lifestyle Behavior Interventions Applying Social Cognitive Theory for Overweight and Obese Girl Adolescents. *Journal of Research in Health Sciences*, *18*(2), e00409.
664534571	Bagherniya, M., Sharma, M., Mostafavi, F., & Keshavarz, S. A. (2015). Application of social cognitive theory in predicting childhood obesity prevention behaviors in overweight and obese Iranian adolescents. *International Quarterly of Community Health Education*, *35*(2), 133–147. https://doi.org/10.1177/0272684X15569487
664539762	Baradaran Mahdavi, S., Bolourinejad, P., Heshmat, R., Motlagh, M. E., Ziaodini, H., Taheri, M., Ahadi, Z., Qorbani, M., & Kelishadi, R. (2019). Association of sedentary leisure time with school performance in children and adolescents: The CASPIAN-V study. *International Journal of Pediatrics*, *Online First*. https://doi.org/10.22038/ijp.2019.38757.3313
664534254	Baradaran Mahdavi, S., Mansourian, M., Shams, E., Qorbani, M., Heshmat, R., Motlagh, M. E., Ziaodini, H., Dashti, R., Taheri, M., & Kelishadi, R. (2020). Association of Sunlight Exposure with Sleep Hours in Iranian Children and Adolescents: The CASPIAN-V Study. *Journal of Tropical Pediatrics*, *66*(1), 4–14. https://doi.org/10.1093/tropej/fmz023
664537420	Bathrellou, E., Lazarou, C., Panagiotakos, D. B., & Sidossis, L. S. (2007). Physical activity patterns and sedentary behaviors of children from urban and rural areas of Cyprus. *Central European Journal of Public Health*, *15*(2), 66–70. https://doi.org/10.21101/cejph.a3414
664535342	Baygi, F., Dorosty, A. R., Kelishadi, R., Qorbani, M., Asayesh, H., Mansourian, M., & Mirkarimi, K. (2012). Determinants of childhood obesity in representative sample of children in north East of iran. *Cholesterol*, *2012*, 875163. https://doi.org/10.1155/2012/875163
664534490	Baygi, F., Heshmat, R., Kelishadi, R., Mohammadi, F., Motlagh, M. E., Ardalan, G., Asayesh, H., Larijani, B., & Qorbani, M. (2015). Regional Disparities in Sedentary Behaviors and Meal Frequency in Iranian Adolescents: The CASPIAN-III Study. *Iranian Journal of Pediatrics*, *25*(2), e182. https://doi.org/10.5812/ijp.182
664538115	Bicer, A. H., & Alsaffar, A. A. (2016). Dietary intake and physical activity levels of male adolescents with autism spectrum disorder (ASD) and normal to high body mass index (BMI) – A case series study. *Research in Autism Spectrum Disorders*, *31*, 1–10. https://doi.org/10.1016/j.rasd.2016.07.002
664810340	Bucksch, J., Sigmundova, D., Hamrik, Z., Troped, P. J., Melkevik, O., Ahluwalia, N., Borraccino, A., Tynjälä, J., Kalman, M., & Inchley, J. (2016). International Trends in Adolescent Screen-Time Behaviors From 2002 to 2010. *The Journal of Adolescent Health: Official Publication of the Society for Adolescent Medicine*, *58*(4), 417–425. https://doi.org/10.1016/j.jadohealth.2015.11.014
664539181	Camliguney, A. F., Mengutay, S., & Pehlivan, A. (2012). Differences in Physical Activity Levels in 8-10 Year-old Girls Who Attend Physical Education Classes Only and Those Who Also Regularly Perform Extracurricular Sports Activities. *Procedia - Social and Behavioral Sciences*, *46*, 4708–4712. https://doi.org/10.1016/j.sbspro.2012.06.323
664537436	Cengiz, C., & Ince, M. L. (2014). Impact of social-ecologic intervention on physical activity knowledge and behaviors of rural students. *Journal of Physical Activity & Health*, *11*(8), 1565–1572. https://doi.org/10.1123/jpah.2013-0080
664810456	Cermak, S. A., Katz, N., Weintraub, N., Steinhart, S., Raz-Silbiger, S., Munoz, M., & Lifshitz, N. (2015). Participation in Physical Activity, Fitness, and Risk for Obesity in Children with Developmental Coordination Disorder: A Cross-cultural Study. *Occupational Therapy International*, *22*(4), 163–173. https://doi.org/10.1002/oti.1393
664537346	Chacar, H. R., & Salameh, P. (2011). Public schools adolescents’ obesity and growth curves in Lebanon. *Le Journal Medical Libanais. The Lebanese Medical Journal*, *59*(2), 80–88.
664539248	Dagci, G., & Saygin, Ö. (2015). Investigation of Physical Activity Levels and Body Compositions of Adolescent Boys and Girls. *Studies on Ethno-Medicine*, *9*(3), 385–390. https://doi.org/10.1080/09735070.2015.11905456
664540105	Daşkapan, A., Şanlı, C., Aydoğan-Arslan, S., Çiledağ-Özdemir, F., Korkem, D., & Kara, U. (2017). Evaluation of the functional capacity, respiratory functions and musculoskeletal systems of the children with chest pain for non-cardiac reasons. *The Turkish Journal of Pediatrics*, *59*(3), 295–303. https://doi.org/10.24953/turkjped.2017.03.010
664537117	Davidsson, L., Al-Ghanim, J., Al-Ati, T., Al-Hamad, N., Al-Mutairi, A., Al-Olayan, L., & Preston, T. (2016). Total Energy Expenditure in Obese Kuwaiti Primary School Children Assessed by the Doubly-Labeled Water Technique. *International Journal of Environmental Research and Public Health*, *13*(10), 1007. https://doi.org/10.3390/ijerph13101007

664537297	De Bourdeaudhuij, I., Verbestel, V., De Henauw, S., Maes, L., Huybrechts, I., Mårild, S., Eiben, G., Moreno, L. A., Barba, G., Kovács, É., Konstabel, K., Tornaritis, M., Gallois, K., Hebestreit, A., Pigeot, I., & IDEFICS consortium. (2015). Behavioural effects of a community-oriented setting-based intervention for prevention of childhood obesity in eight European countries. Main results from the IDEFICS study. *Obesity Reviews: An Official Journal of the International Association for the Study of Obesity*, *16 Suppl 2*, 30–40. https://doi.org/10.1111/obr.12347
664534506	Dianat, I., Alipour, A., & Asghari Jafarabadi, M. (2017). Prevalence and risk factors of low back pain among school age children in Iran. *Health Promotion Perspectives*, *7*(4), 223–229. https://doi.org/10.15171/hpp.2017.39
664539454	Didarloo, A., Sharafkhani, N., Shahnazi, H., Sorkhabi, Z., & sheikhi, S. (2017). Application of Theory of Planned Behavior to improve Obesity-Preventive Lifestyle among Students: A school-based interventional study. *International Journal of Pediatrics*, *Online First*. https://doi.org/10.22038/ijp.2017.24040.2044
664536608	Doaei, S., Jarrahi, S., Torki, S., Haghshenas, R., Jamshidi, Z., Rezaei, S., Moslem, A., Ghorat, F., Khodabakhshi, A., & Gholamalizadeh, M. (2020). Serum vitamin D level may be associated with body weight and body composition in male adolescents; a longitudinal study. *Pediatric Endocrinology, Diabetes, and Metabolism*, *26*(3), 125–131. https://doi.org/10.5114/pedm.2020.97466
664540235	Doğan, M., Derman, O., Oksüz-Kanbur, N., Akgül, S., & Kutluk, T. (2009). The effects of nutrition and physical activity on bone development in male adolescents. *The Turkish Journal of Pediatrics*, *51*(6), 545–550.
664537107	Duncan, M. J., Al-Hazzaa, H. M., Al-Nakeeb, Y., Al-Sobayel, H. I., Abahussain, N. A., Musaiger, A. O., Lyons, M., Collins, P., & Nevill, A. (2014). Anthropometric and lifestyle characteristics of active and inactive Saudi and British adolescents. *American Journal of Human Biology: The Official Journal of the Human Biology Council*, *26*(5), 635–642. https://doi.org/10.1002/ajhb.22574
664534171	Edalati, S., Bagherzadeh, F., Asghari Jafarabadi, M., & Ebrahimi-Mamaghani, M. (2021). Higher ultra-processed food intake is associated with higher DNA damage in healthy adolescents. *The British Journal of Nutrition*, *125*(5), 568–576. https://doi.org/10.1017/S0007114520001981
664535969	Ejtahed, H.-S., Mahmoodi, Z., Qorbani, M., Angoorani, P., Motlagh, M. E., Hasani-Ranjbar, S., Ziaodini, H., Taheri, M., Heshmat, R., & Kelishadi, R. (2021). A comparison between body mass index and waist circumference for identifying continuous metabolic syndrome risk score components in Iranian school-aged children using a structural equation modeling approach: The CASPIAN-V study. *Eating and Weight Disorders: EWD*, *26*(5), 1609–1616. https://doi.org/10.1007/s40519-020-00971-y
664534586	Eliacik, K., Bolat, N., Koçyiğit, C., Kanik, A., Selkie, E., Yilmaz, H., Catli, G., Dundar, N. O., & Dundar, B. N. (2016). Internet addiction, sleep and health-related life quality among obese individuals: A comparison study of the growing problems in adolescent health. *Eating and Weight Disorders: EWD*, *21*(4), 709–717. https://doi.org/10.1007/s40519-016-0327-z
664539209	Elkholy, T., Hassanen, N. H. M., & Rasha. (2012). Demographic, Socio-Economic Factors and Physical Activity Affecting the Nutritional Status of Young Children Under Five Years. *Life Science Journal*, *9*, 3604–3614.
664539071	Erturan, G., McBride, R., & Agbuga, B. (2020). Self-regulation and self-efficacy as mediators of achievement goals and leisure time physical activity: A proposed model. *Pedagogy of Physical Culture and Sports*, *24*(1), 12–20. https://doi.org/10.15561/26649837.2020.0102
664534458	Esatbeyoğlu, F., & KiN İŞler, A. (2018). Physical Activity Levels, BMI and Healthy Life Style Behaviors in Adolescents Living in a Rural District. *Turkiye Klinikleri Journal of Sports Sciences*, *10*(1), 9–18. https://doi.org/10.5336/sportsci.2017-57533
664540012	Esmaeilzadeh, S., & Ebadollahzadeh, K. (2012). Physical fitness, physical activity and sedentary activities of 7 to 11 year old boys with different body mass indexes. *Asian Journal of Sports Medicine*, *3*(2), 105–112. https://doi.org/10.5812/asjsm.34709
664534238	Faridizad, R., Ahadi, Z., Heshmat, R., Motlagh, M. E., Sheidaei, A., Ziaodini, H., Taheri, M., Qorbani, M., Baradaran Mahdavi, S., & Kelishadi, R. (2020). Association of screen time with subjective health complaints in Iranian school-aged children and adolescents: The CASPIAN-V study. *Journal of Public Health*, *28*(1), 31–40. https://doi.org/10.1007/s10389-019-01014-2
664534839	Fazah, A., Jacob, C., Moussa, E., El-Hage, R., Youssef, H., & Delamarche, P. (2010). Activity, inactivity and quality of life among Lebanese adolescents. *Pediatrics International: Official Journal of the Japan Pediatric Society*, *52*(4), 573–578. https://doi.org/10.1111/j.1442-200X.2009.03021.x
664534165	Gencer-Atalay, K., Karadag-Saygi, E., Uzuncakmak, B., Keskin, A., Furtun, Y., Guven, H., Uzunoglu, I., & Kurtel, H. (2021). Daily Physical Activity in Children and Adolescents with Low

	Lumbar and Sacral Level Myelomeningocele. *Developmental Neurorehabilitation*, *24*(3), 145–149. https://doi.org/10.1080/17518423.2020.1800855
664534820	Ghavamzadeh, S., Khalkhali, H. R., & Alizadeh, M. (2013). TV viewing, independent of physical activity and obesogenic foods, increases overweight and obesity in adolescents. *Journal of Health, Population, and Nutrition*, *31*(3), 334–342. https://doi.org/10.3329/jhpn.v31i3.16825
664536907	Ghobadi, S., & Faghih, S. (2018). Eating breakfast and snacks while television viewing are associated with some cardio metabolic risk factors among Iranian children. *Diabetes & Metabolic Syndrome*, *12*(3), 235–243. https://doi.org/10.1016/j.dsx.2017.09.008
664534538	Ghobadi, S., Rostami, Z. H., Marzijarani, M. S., & Faghih, S. (2019). Association of Vitamin D Status and Metabolic Syndrome Components in Iranian Children. *International Journal of Preventive Medicine*, *10*, 77. https://doi.org/10.4103/ijpvm.IJPVM_242_17
664536789	Ghobadi, S., Totosy de Zepetnek, J. O., Hemmatdar, Z., Bellissimo, N., Barati, R., Ahmadnia, H., Salehi-Marzijarani, M., & Faghih, S. (2018). Association between overweight/obesity and eating habits while watching television among primary-school children in the city of Shiraz, Iran. *Public Health Nutrition*, *21*(3), 571–579. https://doi.org/10.1017/S1368980017003251
664540225	Güvenç, A., Açikada, C., Aslan, A., & Ozer, K. (2011). Daily Physical Activity and Physical Fitness in 11-to 15-year-old Trained and Untrained Turkish Boys. *Journal of Sports Science & Medicine*, *10*(3), 502–514.
664540123	Güvenç, A., Aslan, A., & Açıkada, C. (2013). Objectively measured activity in 8-10-year-old Turkish children: Relationship to health-related fitness. *Pediatrics International: Official Journal of the Japan Pediatric Society*, *55*(5), 629–636. https://doi.org/10.1111/ped.12119
664539070	Haghparast, A; Rohani, C; Vasli, P; Salmani, F; Marzaleh, M A. (2020). The Effect of two educational methods of lecturing and peer group on physical activity among 12-15-year-old students in health promoting schools. *Iranian Red Crescent Medical Journal*. https://doi.org/10.32592/ircmj.2020.22.9.59
664534793	Hajian-Tilaki, K., & Heidari, B. (2012). Prevalences of overweight and obesity and their association with physical activity pattern among Iranian adolescents aged 12-17 years. *Public Health Nutrition*, *15*(12), 2246–2252. https://doi.org/10.1017/S1368980012001048
664540034	Hajian-Tilaki, K., & Heidari, B. (2013). Childhood Obesity, Overweight, Socio-Demographic and Life Style Determinants among Preschool Children in Babol, Northern Iran. *Iranian Journal of Public Health*, *42*(11), 1283–1291.
664535051	Hamaideh, S. H., Al-Khateeb, R. Y., & Al-Rawashdeh, A. B. (2010). Overweight and obesity and their correlates among Jordanian adolescents. *Journal of Nursing Scholarship: An Official Publication of Sigma Theta Tau International Honor Society of Nursing*, *42*(4), 387–394. https://doi.org/10.1111/j.1547-5069.2010.01367.x
664536282	Hashem, R., Rey-López, J. P., Hamer, M., McMunn, A., Whincup, P. H., Owen, C. G., Rowlands, A., & Stamatakis, E. (2018). Physical Activity and Sedentary Behaviors Levels of Kuwaiti Adolescents: The Study of Health and Activity Among Adolescents in Kuwait. *Journal of Physical Activity & Health*, *15*(4), 255–262. https://doi.org/10.1123/jpah.2017-0013
664536181	Hashem, R., Rey-López, J. P., Hamer, M., McMunn, A., Rowlands, A., Whincup, P. H., Owen, C. G., Ding, D., Powell, L., & Stamatakis, E. (2019). Associations between objectively assessed and questionnaire-based sedentary behaviour with body mass index and systolic blood pressure in Kuwaiti adolescents. *BMC Research Notes*, *12*(1), 588. https://doi.org/10.1186/s13104-019-4626-0
664537990	Hassan, M. O., & Al-Kharusy, W. (2000). Physical fitness and fatness among Omani schoolboys: A pilot study. *Journal for Scientific Research. Medical Sciences*, *2*(1), 37–41.
664538353	Hawi, N. S., & Rupert, M. S. (2015). Impact of e-Discipline on Children's Screen Time. *Cyberpsychology, Behavior and Social Networking*, *18*(6), 337–342. https://doi.org/10.1089/cyber.2014.0608
664537091	Henry, C. J. K., Lightowler, H. J., & Al-Hourani, H. M. (2004). Physical activity and levels of inactivity in adolescent females ages 11-16 years in the United Arab Emirates. *American Journal of Human Biology: The Official Journal of the Human Biology Council*, *16*(3), 346–353. https://doi.org/10.1002/ajhb.20022
664536952	Hovsepian, S., Kelishadi, R., Motlagh, M. E., Kasaeian, A., Shafiee, G., Arefirad, T., Najafi, F., Khoramdad, M., Asayesh, H., Heshmat, R., & Qorbani, M. (2016). Level of physical activity and screen time among Iranian children and adolescents at the national and provincial level: The CASPIAN-IV study. *Medical Journal of the Islamic Republic of Iran*, *30*, 422.
664534507	Jalali-Farahani, S., Amiri, P., & Chin, Y. S. (2016). Are physical activity, sedentary behaviors and sleep duration associated with body mass index-for-age and health-related quality of life among high

	school boys and girls? *Health and Quality of Life Outcomes*, *14*, 30. https://doi.org/10.1186/s12955-016-0434-6
664536319	Jalilpiran, Y., Mozaffari, H., Askari, M., Jafari, A., & Azadbakht, L. (2021). The association between Healthy Beverage Index and anthropometric measures among children: A cross-sectional study. *Eating and Weight Disorders: EWD*, *26*(5), 1437–1445. https://doi.org/10.1007/s40519-020-00954-z
664536692	Kalantari, N., Keshavarz Mohammadi, N., Izadi, P., Gholamalizadeh, M., Doaei, S., Eini-Zinab, H., Salonurmi, T., Rafieifar, S., Janipoor, R., & Azizi Tabesh, G. (2018). A complete linkage disequilibrium in a haplotype of three SNPs in Fat Mass and Obesity associated (FTO) gene was strongly associated with anthropometric indices after controlling for calorie intake and physical activity. *BMC Medical Genetics*, *19*(1), 146. https://doi.org/10.1186/s12881-018-0664-z
664537699	Karaca, A., Caglar, E., Bilgili, N., & Ayaz, S. (2011). Screen time of adolescents in an economically developing country: The case of Turkey. *Annals of Human Biology*, *38*(1), 28–33. https://doi.org/10.3109/03014460.2010.486383
664535079	Kelishadi, R., Ardalan, G., Gheiratmand, R., Gouya, M. M., Razaghi, E. M., Delavari, A., Majdzadeh, R., Heshmat, R., Motaghian, M., Barekati, H., Mahmoud-Arabi, M. S., Riazi, M. M., & CASPIAN Study Group. (2007). Association of physical activity and dietary behaviours in relation to the body mass index in a national sample of Iranian children and adolescents: CASPIAN Study. *Bulletin of the World Health Organization*, *85*(1), 19–26. https://doi.org/10.2471/blt.06.030783
664536613	Kelishadi, R., Heidari, Z., Kazemi, I., Jafari-Koshki, T., Mansourian, M., Motlagh, M.-E., & Heshmat, R. (2018). A hierarchical Bayesian tri-variate analysis on factors associated with anthropometric measures in a large sample of children and adolescents: The CASPIAN-IV study. *Journal of Pediatric Endocrinology & Metabolism: JPEM*, *31*(4), 443–449. https://doi.org/10.1515/jpem-2017-0213
664537902	Kelishadi, R., Mirghaffari, N., Poursafa, P., & Gidding, S. S. (2009). Lifestyle and environmental factors associated with inflammation, oxidative stress and insulin resistance in children. *Atherosclerosis*, *203*(1), 311–319. https://doi.org/10.1016/j.atherosclerosis.2008.06.022
664537407	Kelishadi, R., Razaghi, E. M., Gouya, M. M., Ardalan, G., Gheiratmand, R., Delavari, A., Motaghian, M., Ziaee, V., Siadat, Z. D., Majdzadeh, R., Heshmat, R., Barekati, H., Arabi, M. S. M., Heidarzadeh, A., Shariatinejad, K., & CASPIAN Study Group. (2007). Association of physical activity and the metabolic syndrome in children and adolescents: CASPIAN Study. *Hormone Research*, *67*(1), 46–52. https://doi.org/10.1159/000096121
664535640	Kerkadi, A., Sadig, A. H., Bawadi, H., Al Thani, A. A. M., Al Chetachi, W., Akram, H., Al-Hazzaa, H. M., & Musaiger, A. O. (2019). The Relationship between Lifestyle Factors and Obesity Indices among Adolescents in Qatar. *International Journal of Environmental Research and Public Health*, *16*(22), 4428. https://doi.org/10.3390/ijerph16224428
664539702	Khader, Y., Irshaidat, O., Khasawneh, M., Amarin, Z., Alomari, M., & Batieha, A. (2009). Overweight and obesity among school children in Jordan: Prevalence and associated factors. *Maternal and Child Health Journal*, *13*(3), 424–431. https://doi.org/10.1007/s10995-008-0362-0
664540155	Khayyatzadeh, S. S., Vatanparast, H., Avan, A., Bagherniya, M., Bahrami, A., Kiani, M. A., Bahrami-Taghanaki, H., Ferns, G. A., & Ghayour-Mobarhan, M. (2017). Serum Transaminase Concentrations and the Presence of Irritable Bowel Syndrome Are Associated with Serum 25-Hydroxy Vitamin D Concentrations in Adolescent Girls Who Are Overweight and Obese. *Annals of Nutrition & Metabolism*, *71*(3–4), 234–241. https://doi.org/10.1159/000484634
664534912	Khoo, S., & Al-Shamli, A. K. (2012). Leisure-time physical activity and physical fitness of male adolescents in Oman. *Asia-Pacific Journal of Public Health*, *24*(1), 128–135. https://doi.org/10.1177/1010539510366178
664537127	Kilani, H., Al-Hazzaa, H., Waly, M. I., & Musaiger, A. (2013). Lifestyle Habits: Diet, physical activity and sleep duration among Omani adolescents. *Sultan Qaboos University Medical Journal*, *13*(4), 510–519. https://doi.org/10.12816/0003309
664535072	Kin-Isler, A., Asci, F. H., Altintas, A., & Guven-Karahan, B. (2009). Physical activity levels and patterns of 11-14 year-old Turkish adolescents. *Adolescence*, *44*(176), 1005–1015.
664540170	Koçak, S., Harris, M. B., Kin İşler, A., & Çiçek, Ş. (2002). Physical Activity Level, Sport Participation, and Parental Education Level in Turkish Junior High School Students. *Pediatric Exercise Science*, *14*(2), 147–154. https://doi.org/10.1123/pes.14.2.147
664537698	Kocaoglu, B., Moschonis, G., Dimitriou, M., Kolotourou, M., Keskin, Y., Sur, H., Hayran, O., & Manios, Y. (2005). Parental educational level and cardiovascular disease risk factors in schoolchildren in large urban areas of Turkey: Directions for public health policy. *BMC Public Health*, *5*, 13. https://doi.org/10.1186/1471-2458-5-13

664537065	Kondolot, M., Poyrazoğlu, S., Horoz, D., Borlu, A., Altunay, C., Balcı, E., Öztürk, A., Mazıcıoğlu, M. M., & Kurtoğlu, S. (2017). Risk factors for overweight and obesity in children aged 2-6 years. *Journal of Pediatric Endocrinology & Metabolism: JPEM*, *30*(5), 499–505. https://doi.org/10.1515/jpem-2016-0358
664540264	Kovács, E., Siani, A., Konstabel, K., Hadjigeorgiou, C., de Bourdeaudhuij, I., Eiben, G., Lissner, L., Gwozdz, W., Reisch, L., Pala, V., Moreno, L. A., Pigeot, I., Pohlabeln, H., Ahrens, W., Molnár, D., & IDEFICS consortium. (2014). Adherence to the obesity-related lifestyle intervention targets in the IDEFICS study. *International Journal of Obesity (2005)*, *38 Suppl 2*(Suppl 2), S144-151. https://doi.org/10.1038/ijo.2014.145
664537221	Lazarou, C., & Matalas, A.-L. (2015). Breakfast intake is associated with nutritional status, Mediterranean diet adherence, serum iron and fasting glucose: The CYFamilies study. *Public Health Nutrition*, *18*(7), 1308–1316. https://doi.org/10.1017/S1368980014001967
664537651	Lazarou, C., Panagiotakos, D. B., Kouta, C., & Matalas, A.-L. (2009). Dietary and other lifestyle characteristics of Cypriot school children: Results from the nationwide CYKIDS study. *BMC Public Health*, *9*, 147. https://doi.org/10.1186/1471-2458-9-147
664538100	Loucaides, C. A. (2018). Seasonal differences in segmented-day physical activity and sedentary behaviour in primary school children. *Early Child Development and Care*, *188*(3), 410–420. https://doi.org/10.1080/03004430.2016.1226292
664538311	Loucaides, C. A. (2020). Screen time behaviour in a sample of 11- to 12-year-old Greek-Cypriot children: A cross-sectional study of parental and child reports. *SAGE Open Medicine*, *8*, 205031212094623. https://doi.org/10.1177/2050312120946232
664540077	Loucaides, C. A., Chedzoy, S. M., & Bennett, N. (2004). Differences in physical activity levels between urban and rural school children in Cyprus. *Health Education Research*, *19*(2), 138–147. https://doi.org/10.1093/her/cyg014
664537900	Loucaides, C. A., & Jago, R. (2006). Correlates of Pedometer-Assessed Physical Activity in Cypriot Elementary School Children. *Journal of Physical Activity & Health*, *3*(3), 267–276. https://doi.org/10.1123/jpah.3.3.267
664540144	Loucaides, C. A., & Jago, R. (2008). Differences in physical activity by gender, weight status and travel mode to school in Cypriot children. *Preventive Medicine*, *47*(1), 107–111. https://doi.org/10.1016/j.ypmed.2008.01.025
664540274	Loucaides, C. A., Jago, R., & Theophanous, M. (2011). Physical activity and sedentary behaviours in Greek-Cypriot children and adolescents: A cross-sectional study. *The International Journal of Behavioral Nutrition and Physical Activity*, *8*, 90. https://doi.org/10.1186/1479-5868-8-90
664537880	Manios, Y., Dimitriou, M., Moschonis, G., Kocaoglu, B., Sur, H., Keskin, Y., & Hayran, O. (2004). Cardiovascular disease risk factors among children of different socioeconomic status in Istanbul, Turkey: Directions for public health and nutrition policy. *Lipids in Health and Disease*, *3*, 11. https://doi.org/10.1186/1476-511X-3-11
664535170	Manios, Y., Kolotourou, M., Moschonis, G., Sur, H., Keskin, Y., Kocaoglu, B., & Hayran, O. (2005). Macronutrient intake, physical activity, serum lipids and increased body weight in primary schoolchildren in Istanbul. *Pediatrics International: Official Journal of the Japan Pediatric Society*, *47*(2), 159–166. https://doi.org/10.1111/j.1442-200x.2005.02047.x
664539125	Marashi, T., Safari-Moradabadi, A., Ahmadi, F., & Alipour-Anbarani, M. (2022). The effect of education based on the theory of planned behavior on the promotion of physical activity and knowledge of students about diabetes prevention. *International Journal of Health Promotion and Education*, *60*(6), 316–328. https://doi.org/10.1080/14635240.2020.1854814
664534252	Mehranfar, S., Jalilpiran, Y., Surkan, P. J., & Azadbakht, L. (2020). Association between protein-rich dietary patterns and anthropometric measurements among children aged 6 years. *Nutrition & Dietetics: The Journal of the Dietitians Association of Australia*, *77*(3), 359–367. https://doi.org/10.1111/1747-0080.12609
664536584	Miri, S. F., Javadi, M., Lin, C.-Y., Griffiths, M. D., Björk, M., & Pakpour, A. H. (2019). Effectiveness of cognitive-behavioral therapy on nutrition improvement and weight of overweight and obese adolescents: A randomized controlled trial. *Diabetes & Metabolic Syndrome*, *13*(3), 2190–2197. https://doi.org/10.1016/j.dsx.2019.05.010
664534164	Mohammadifard, N., Mahdavi, A., Khosravi, A., Esmaillzadeh, A., Feizi, A., & Sarrafzadegan, N. (2021). Salt intake and its sources in children, adolescents and adults in the Islamic Republic of Iran. *Eastern Mediterranean Health Journal = La Revue De Sante De La Mediterranee Orientale = Al-Majallah Al-Sihhiyah Li-Sharq Al-Mutawassit*, *27*(3), 279–286. https://doi.org/10.26719/2021.27.3.279

664534921	Mohseni-Takalloo, S., Mirmiran, P., Hosseini-Esfahani, F., Mehrabi, Y., & Azizi, F. (2014). Metabolic Syndrome and its Association with Healthy Eating Index-2005 in Adolescents: Tehran Lipid and Glucose Study. *Journal of Food and Nutrition Research*, *2*(4), 155–161. https://doi.org/10.12691/jfnr-2-4-4
664539049	Moludi, J., Ebrahimi, B., Maleki, V., Saiedi, S., Tandoroost, A., Jafari-Vayghyan, H., Alizadeh, S., & Djafarian, K. (2020). Comparison of dietary macro and micronutrient intake with physical activity levels among children with and without autism: A case-control study. *Progress in Nutrition*, *21*(2-S), 49–55. https://doi.org/10.23751/pn.v21i2-S.6578
664534207	Moradi, G., Mostafavi, F., Piroozi, B., Zareie, B., Mahboobi, M., & Rasouli, M. A. (2020). The prevalence of physical inactivity in Iranian adolescents and the impact of economic and social inequalities on it: Results of a National Study in 2018. *BMC Public Health*, *20*(1), 1499. https://doi.org/10.1186/s12889-020-09618-0
664534598	Moradi-Lakeh, M., El Bcheraoui, C., Tuffaha, M., Daoud, F., Al Saeedi, M., Basulaiman, M., Memish, Z. A., Al Mazroa, M. A., Al Rabeeah, A. A., & Mokdad, A. H. (2016). The health of Saudi youths: Current challenges and future opportunities. *BMC Family Practice*, *17*, 26. https://doi.org/10.1186/s12875-016-0425-z
664534903	Motamed-Gorji, N., Qorbani, M., Nikkho, F., Asadi, M., Motlagh, M. E., Safari, O., Arefirad, T., Asayesh, H., Mohammadi, R., Mansourian, M., & Kelishadi, R. (2019). Association of screen time and physical activity with health-related quality of life in Iranian children and adolescents. *Health and Quality of Life Outcomes*, *17*(1), 2. https://doi.org/10.1186/s12955-018-1071-z
664538336	Mozafarian, N., Kelishadi, R., Motlagh, M. E., & Maracy, M. (2016). Propensity Score Application in the Relationship of Screen Time and Metabolic Syndrome in Adolescents: The CASPIAN-III Study. *International Journal of Pediatrics*, *4*(3). https://doi.org/10.22038/ijp.2016.6519
664535260	Mozaffari, H., & Nabaei, B. (2007). Obesity and related risk factors. *Indian Journal of Pediatrics*, *74*(3), 265–267.
664534612	Musaiger, A. O., Al-Mufty, B. A., & Al-Hazzaa, H. M. (2014). Eating habits, inactivity, and sedentary behavior among adolescents in Iraq: Sex differences in the hidden risks of noncommunicable diseases. *Food and Nutrition Bulletin*, *35*(1), 12–19. https://doi.org/10.1177/156482651403500102
664537198	Najafabadi, M. G., Memari, A.-H., Kordi, R., Shayestehfar, M., & Eshghi, M.-A. (2017). Mental training can improve physical activity behavior in adolescent girls. *Journal of Sport and Health Science*, *6*(3), 327–332. https://doi.org/10.1016/j.jshs.2015.09.011
664534221	Naseri, P., Amiri, P., Momenyan, S., Zayeri, F., Karimi, M., & Azizi, F. (2020). Longitudinal association between body mass index and physical activity among adolescents with different parental risk: A parallel latent growth curve modeling approach. *The International Journal of Behavioral Nutrition and Physical Activity*, *17*(1), 59. https://doi.org/10.1186/s12966-020-00961-4
664537271	Nasreddine, L., Naja, F., Akl, C., Chamieh, M. C., Karam, S., Sibai, A.-M., & Hwalla, N. (2014). Dietary, lifestyle and socio-economic correlates of overweight, obesity and central adiposity in Lebanese children and adolescents. *Nutrients*, *6*(3), 1038–1062. https://doi.org/10.3390/nu6031038
664536972	Nejadsadeghi, E., Sadeghi, R., Shojaeizadeh, D., Yekaninejad, M. S., Djazayeri, A., & Majlesi, F. (2018). Influence of lifestyle factors on Body Mass Index in preschoolers in Behbahan city, southwest Iran, 2016. *Electronic Physician*, *10*(4), 6725–6732. https://doi.org/10.19082/6725
664810889	Nemet, D., Barkan, S., Epstein, Y., Friedland, O., Kowen, G., & Eliakim, A. (2005). Short- and long-term beneficial effects of a combined dietary-behavioral-physical activity intervention for the treatment of childhood obesity. *Pediatrics*, *115*(4), e443-449. https://doi.org/10.1542/peds.2004-2172
664810468	Nemet, D., Levi, L., Pantanowitz, M., & Eliakim, A. (2014). A combined nutritional-behavioral-physical activity intervention for the treatment of childhood obesity—A 7-year summary. *Journal of Pediatric Endocrinology & Metabolism: JPEM*, *27*(5–6), 445–451. https://doi.org/10.1515/jpem-2013-0349
664536956	Neshatbini Tehrani, A., Farhadnejad, H., Salehpour, A., Beyzai, B., Hekmatdoost, A., & Rashidkhani, B. (2019). The association between nutrition knowledge and adherence to a Mediterranean dietary pattern in Iranian female adolescents. *International Journal of Adolescent Medicine and Health*, *33*(4). https://doi.org/10.1515/ijamh-2018-0188
664534744	Ng, S. W., Zaghloul, S., Ali, H., Harrison, G., Yeatts, K., El Sadig, M., & Popkin, B. M. (2011). Nutrition transition in the United Arab Emirates. *European Journal of Clinical Nutrition*, *65*(12), 1328–1337. https://doi.org/10.1038/ejcn.2011.135
664535087	Nitzan Kaluski, D., Demem Mazengia, G., Shimony, T., Goldsmith, R., & Berry, E. M. (2009). Prevalence and determinants of physical activity and lifestyle in relation to obesity among

	schoolchildren in Israel. *Public Health Nutrition*, *12*(6), 774–782. https://doi.org/10.1017/S1368980008002991
664539221	Nozari, R. A., Farshbaf-Khalili, A., Sattarzadeh, N., & Jafarabadi, M. A. (2019). The Effect of Counseling on Menstrual Hygiene, Physical Activity, and Nutritional Status of Female Adolescent Students: A Randomized Controlled Field Trial. *Crescent Journal of Medical and Biological Sciences*, *6*(3), 393–402.
664536833	Okur, E. O., Inal-Ince, D., Saglam, M., Vardar-Yagli, N., & Arikan, H. (2021). Physical activity patterns in children with cerebral palsy and typically developing peers. *Physiotherapy Theory and Practice*, *37*(6), 710–718. https://doi.org/10.1080/09593985.2019.1641863
664538809	Olafsdottir, S., Berg, C., Eiben, G., Lanfer, A., Reisch, L., Ahrens, W., Kourides, Y., Molnár, D., Moreno, L. A., Siani, A., Veidebaum, T., & Lissner, L. (2014). Young children's screen activities, sweet drink consumption and anthropometry: Results from a prospective European study. *European Journal of Clinical Nutrition*, *68*(2), 223–228. https://doi.org/10.1038/ejcn.2013.234
664538308	Ozmert, E. N., Ozdemir, R., Pektaş, A., Uçkardeş, Y., & Yurdakök, K. (2011). Effect of activity and television viewing on BMI z-score in early adolescents in Turkey. *World Journal of Pediatrics: WJP*, *7*(1), 37–40. https://doi.org/10.1007/s12519-011-0243-2
664810851	Pinhas-Hamiel, O., Bar-Zvi, E., Boyko, V., Reichman, B., & Lerner-Geva, L. (2009). Prevalence of overweight in kindergarten children in the centre of Israel—Association with lifestyle habits. *Child: Care, Health and Development*, *35*(2), 147–152. https://doi.org/10.1111/j.1365-2214.2008.00905.x
664534826	Platat, C., & Jarrar, A. (2012). Reliability and validity of a physical activity questionnaire in children. *International Journal of Food Sciences and Nutrition*, *63*(6), 637–644. https://doi.org/10.3109/09637486.2011.644766
664539029	Qahwaji, D. (2012). Physical activity and life style among Male Adolescents in Jeddah, Saudi Arabia. *Life Science Journal*, *9*, 1163–1172.
664534478	Rahimi, H., Yuzbashian, E., Zareie, R., Asghari, G., Djazayery, A., Movahedi, A., & Mirmiran, P. (2020). Dietary approaches to stop hypertension (DASH) score and obesity phenotypes in children and adolescents. *Nutrition Journal*, *19*(1), 112. https://doi.org/10.1186/s12937-020-00631-y
664810637	Raz-Silbiger, S., Lifshitz, N., Katz, N., Steinhart, S., Cermak, S. A., & Weintraub, N. (2015). Relationship between motor skills, participation in leisure activities and quality of life of children with Developmental Coordination Disorder: Temporal aspects. *Research in Developmental Disabilities*, *38*, 171–180. https://doi.org/10.1016/j.ridd.2014.12.012
664537025	Rostamian, M., & Kazemi, A. (2016). Relationship between observational learning and health belief with physical activity among adolescents girl in Isfahan, Iran. *Iranian Journal of Nursing and Midwifery Research*, *21*(6), 601–604. https://doi.org/10.4103/1735-9066.197681
664539146	Rostami-Moez, M., Hazavehei, S. M. M., Karami, M., Karimi-Shahanjarini, A., Nazem, F., & Rezapur-Shahkolai, F. (2017). Decline in Physical Activity Among Iranian Girl Students Aged 10 to 16 and the Related Factors. *Health Scope*, *In Press*(In Press). https://doi.org/10.5812/jhealthscope.62422
664534732	Safiri, S., Kelishadi, R., Qorbani, M., Abbasi-Ghah-Ramanloo, A., Motlagh, M. E., Ardalan, G., Shafiee, G., Ahadi, Z., Sanaei, M., Asayesh, H., Larijani, B., & Heshmat, R. (2015). Screen time and its relation to cardiometabolic risk among children and adolescents: The CASPIAN-III study. *Iranian Journal of Public Health*, *44*(1), 35–44.
664539589	Saglam, D., Samur, G., & Turan, S. (2019). Assessment of vitamin D status in turkish adolescents: Its relation to obesity, cardiometabolic risk factors and nutritional status. *Progress in Nutrition*, *21*(4), 762–768. https://doi.org/10.23751/pn.v21i4.6850
664537759	Salamoun, M. M., Kizirian, A. S., Tannous, R. I., Nabulsi, M. M., Choucair, M. K., Deeb, M. E., & El-Hajj Fuleihan, G. A. (2005). Low calcium and vitamin D intake in healthy children and adolescents and their correlates. *European Journal of Clinical Nutrition*, *59*(2), 177–184. https://doi.org/10.1038/sj.ejcn.1602056
664534616	Sanaeinasab, H., Saffari, M., Nazeri, M., Karimi Zarchi, A., & Cardinal, B. J. (2013). Descriptive analysis of Iranian adolescents' stages of change for physical activity behavior. *Nursing & Health Sciences*, *15*(3), 280–285. https://doi.org/10.1111/nhs.12020
664535071	Sanaeinasab, H., Tavakoli, R., Karimizarchi, A., Amini, Z. H., Farokhian, A., & Najarkolaei, F. R. (2014). The effectiveness of education using the health belief model in preventing osteoporosis among female students. *Eastern Mediterranean Health Journal = La Revue De Sante De La Mediterranee Orientale = Al-Majallah Al-Sihhiyah Li-Sharq Al-Mutawassit*, *19 Suppl 3*, S38-44.
664534214	Sarsour, A., Turban, M., Al Wahaidi, A., Abed, Y., & Alkahlout, H. (2019). Does gender influence food intake and physical activity pattern among Palestinian adolescents in the Gaza Strip? *Eastern*

	Mediterranean Health Journal = La Revue De Sante De La Mediterranee Orientale = Al-Majallah Al-Sihhiyah Li-Sharq Al-Mutawassit, *25*(10), 722–727. https://doi.org/10.26719/emhj.19.073
664539193	Saygin, O. (2014). Physical Activity Level and Obesity Prevalence of Primary and Secondary Students. *The Anthropologist*, *18*(2), 371–377. https://doi.org/10.1080/09720073.2014.11891555
664537646	Saygin, O., Zorba, E., Karacabey, K., & Mengutay, S. (2007). Gender and maturation differences in health-related physical fitness and physical activity in Turkish children. *Pakistan Journal of Biological Sciences: PJBS*, *10*(12), 1963–1969. https://doi.org/10.3923/pjbs.2007.1963.1969
664540060	Sedaghat, F., Naja, F., Darand, M., Beyzai, B., & Rashidkhani, B. (2017). Adherence to a Mediterranean dietary pattern and overweight and obesity among female adolescents in Iran. *International Journal of Adolescent Medicine and Health*, *31*(2), /j/ijamh.2019.31.issue-2/ijamh-2016-0160/ijamh-2016-0160.xml. https://doi.org/10.1515/ijamh-2016-0160
664810396	Sherman, G., Nemet, D., Moshe, V., Consolaro, A., Ravelli, A., Ruperto, N., Uziel, Y., & Paediatric Rheumatology International Trials Organisation (PRINTO). (2018). Disease activity, overweight, physical activity and screen time in a cohort of patients with juvenile idiopathic arthritis. *Clinical and Experimental Rheumatology*, *36*(6), 1110–1116.
664535015	Shokrvash, B., Majlessi, F., Montazeri, A., Nedjat, S., Rahimi, A., Djazayeri, A., & Shojaeezadeh, D. (2013). Correlates of physical activity in adolescence: A study from a developing country. *Global Health Action*, *6*, 20327. https://doi.org/10.3402/gha.v6i0.20327
664534653	Simsek, S., Inal-Ince, D., Cakmak, A., Emiralioglu, N., Calik-Kutukcu, E., Saglam, M., Vardar-Yagli, N., Ozcelik, H. U., Sonbahar-Ulu, H., Bozdemir-Ozel, C., Kiper, N., & Arikan, H. (2018). Reduced anaerobic and aerobic performance in children with primary ciliary dyskinesia. *European Journal of Pediatrics*, *177*(5), 765–773. https://doi.org/10.1007/s00431-018-3121-2
664534160	Smith, E., Fazeli, F., Wilkinson, K., & Clark, C. C. T. (2021). Physical behaviors and fundamental movement skills in British and Iranian children: An isotemporal substitution analysis. *Scandinavian Journal of Medicine & Science in Sports*, *31*(2), 398–404. https://doi.org/10.1111/sms.13837
664540021	Soltanian, A. R., Amiri, M., Namazi, S., Qaedi, H., & Kohan, G. R. (2014). Mental Health Changes and Its Predictors in Adolescents using the Path Analytic Model: A 7-Year Observational Study. *Iranian Journal of Psychiatry*, *9*(1), 1–7.
664538642	Sourtiji, H., Hosseini, S. A., Rassafiani, M., Kohan, A., Noroozi, M., & Motlagh, M. E. (2018). The Associations Between Screen Time, Sleep Duration, and Body Mass Index (BMI) in Under Five-Year-Old Children. *Archives of Neuroscience*, *In Press*(In Press). https://doi.org/10.5812/ans.81229
664535168	Sur, H., Kolotourou, M., Dimitriou, M., Kocaoglu, B., Keskin, Y., Hayran, O., & Manios, Y. (2005). Biochemical and behavioral indices related to BMI in schoolchildren in urban Turkey. *Preventive Medicine*, *41*(2), 614–621. https://doi.org/10.1016/j.ypmed.2004.11.029
664537660	Tarakci, E., Yeldan, I., Kaya Mutlu, E., Baydogan, S. N., & Kasapcopur, O. (2011). The relationship between physical activity level, anxiety, depression, and functional ability in children and adolescents with juvenile idiopathic arthritis. *Clinical Rheumatology*, *30*(11), 1415–1420. https://doi.org/10.1007/s10067-011-1832-0
664538202	Taymoori, P., & Lubans, D. R. (2008). Mediators of behavior change in two tailored physical activity interventions for adolescent girls. *Psychology of Sport and Exercise*, *9*(5), 605–619. https://doi.org/10.1016/j.psychsport.2007.09.001
664535123	Taymoori, P., Niknami, S., Berry, T., Ghofranipour, F., & Kazemnejad, A. (2009). Application of the health promotion model to predict stages of exercise behaviour in Iranian adolescents. *Eastern Mediterranean Health Journal = La Revue de Santé de La Méditerranée Orientale = Al-Majallah al-Ṣiḥḥīyah Li-Sharq al-Mutawassiṭ*, *15*, 1215–1225.
664534966	Taymoori, P., Rhodes, R. E., & Berry, T. R. (2010). Application of a social cognitive model in explaining physical activity in Iranian female adolescents. *Health Education Research*, *25*(2), 257–267. https://doi.org/10.1093/her/cyn051
664534914	Taymoori, P., Berry, T. R., & Lubans, D. R. (2012). Tracking of physical activity during middle school transition in Iranian adolescents. *Health Education Journal*, *71*(6), 631–641. https://doi.org/10.1177/0017896911419341
664540166	Teber, S., Saglam, M., Ertugrul, I., Vardar-Yagli, N., Cakmak, A., Calik-Kutukcu, E., Inal-Ince, D., Arikan, H., & Karagoz, T. (2020). Levels of Physical Activity and Physical Fitness in Pediatric Pacemaker Patients: A Cross-Sectional Study. *Pediatric Cardiology*, *41*(7), 1363–1369. https://doi.org/10.1007/s00246-020-02384-1
664540008	Tayyem, R., Al-Hazzaa, H., Abumweis, S., Bawadi, H., Musaiger, A., & Qatatsheh, A. (2014). Association of Lifestyle Factors with Obesity Indices among Adolescents Living in Amman. *Malaysian Journal of Nutrition*, *20*, 51–62.

664540006	Tayyem, R. F., Al-Hazzaa, H. M., Abu-Mweis, S. S., Bawadi, H. A., Hammad, S. S., [illegible] Musaiger, A. O. (2014). Dietary habits and physical activity levels in Jordanian adolescents attending private versus public schools. *Eastern Mediterranean Health Journal = La Revue De Sante De La Mediterranee Orientale = Al-Majallah Al-Sihhiyah Li-Sharq Al-Mutawassit*, *20*(7), 416–423.
664537093	Yiallouros, P. K., Economou, M., Kolokotroni, O., Savva, S. C., Gavatha, M., Ioannou, P., Karpathios, T., & Middleton, N. (2015). Gender differences in objectively assessed physical activity in asthmatic and non-asthmatic children. *Pediatric Pulmonology*, *50*(4), 317–326. https://doi.org/10.1002/ppul.23045
664537295	Yilmaz, G., Demirli Caylan, N., & Karacan, C. D. (2015). An intervention to preschool children for reducing screen time: A randomized controlled trial. *Child: Care, Health and Development*, *41*(3), 443–449. https://doi.org/10.1111/cch.12133
664539158	Yurdakul, H. Ö., & Baydemir, B. (2020). Comparison of physical activity and skinfold thickness of students living in rural and city center. *Pedagogy of Physical Culture and Sports*, *24*(5), 271–277. https://doi.org/10.15561/26649837.2020.0508

Table S5. Extracted PA and SB data from all studies identified.

Notes: All data were extracted that met the definition of daily volume of PA or SB. As far as possible the results are reported in terms of the original paper's terminology.

See S4 for a full list of the references used.

<Excel spreadsheet>
